# Tumor-infiltrating lymphocytes in cancer immunotherapy: from chemotactic recruitment to translational modeling

**DOI:** 10.3389/fimmu.2025.1601773

**Published:** 2025-05-22

**Authors:** Fatjona Pupuleku Kraja, Vladimir B. Jurisic, Altijana Hromić-Jahjefendić, Nafsika Rossopoulou, Theodora Katsila, Katarina Mirjacic Martinovic, Javier De Las Rivas, Carmen Cristina Diaconu, Árpád Szöőr

**Affiliations:** ^1^ Oncology Clinic, University Hospital Center Mother Teresa, Tirana, Albania; ^2^ Institute of Pathophysiology, Faculty of Medical Sciences, University of Kragujevac, Kragujevac, Serbia; ^3^ Department of Genetics and Bioengineering, Faculty of Engineering and Natural Sciences, International University of Sarajevo, Sarajevo, Bosnia and Herzegovina; ^4^ Institute of Chemical Biology, National Hellenic Research Foundation, Athens, Greece; ^5^ Laboratory of Immunology, Department of Experimental Oncology, Institute of Oncology and Radiology of Serbia, Belgrade, Serbia; ^6^ Bioinformatics and Functional Genomics Group, Cancer Research Center (CiC-IBMCC, CSIC/USAL), Consejo Superior de Investigaciones Cientificas (CSIC) and University of Salamanca (USAL), Salamanca, Spain; ^7^ Department of Cellular and Molecular Pathology, Stefan S. Nicolau Institute of Virology, Bucharest, Romania; ^8^ Department of Biophysics and Cell Biology, Faculty of Medicine, University of Debrecen, Debrecen, Hungary

**Keywords:** tumor-infiltrating lymphocytes, tumor microenvironment, immunotherapy, adoptive cell transfer, experimental models

## Abstract

Tumor-infiltrating lymphocytes (TILs) are a diverse population of immune cells that play a central role in tumor immunity and have emerged as critical mediators in cancer immunotherapy. This review explores the phenotypic and functional diversity of TILs—including CD8^+^ cytotoxic T cells, CD4^+^ helper T cells, regulatory T cells, B cells, and natural killer (NK) cells—and their dynamic interactions within the tumor microenvironment (TME). While TILs can drive tumor regression, their activity is often hindered by immune checkpoint signaling, metabolic exhaustion, and stromal exclusion. We highlight TIL recruitment, activation, and polarization mechanisms, focusing on chemokine gradients, endothelial adhesion molecules, and dendritic cell-mediated priming. Special emphasis is placed on preclinical models that evaluate TIL function, including 3D tumor spheroids, organoid co-cultures, syngeneic mouse models, and humanized systems. These provide valuable platforms for optimizing TIL-based therapies. Furthermore, we examine the prognostic and predictive value of TILs across cancer types, their role in adoptive cell therapy, and the challenges of translating preclinical success into clinical efficacy. Emerging technologies such as single-cell sequencing, neoantigen prediction, and biomaterial platforms are transforming our understanding of TIL biology and enhancing their therapeutic potential. Innovative strategies—ranging from genetic engineering and combination therapies to targeted modulation of the TME—are being developed to overcome resistance mechanisms and improve TIL persistence, infiltration, and cytotoxicity. This review integrates current advances in TIL research and therapy, offering a comprehensive foundation for future clinical translation. TILs hold significant promise as both biomarkers and therapeutic agents, and with continued innovation, they are poised to become a cornerstone of personalized cancer immunotherapy.

## 1 Introduction

Tumor-infiltrating lymphocytes (TILs) represent a crucial component of the tumor microenvironment (TME), playing a pivotal role in tumor immunity and influencing cancer progression (1). TILs are a diverse group of immune cells that infiltrate tumor tissues, and their role can be both proinflammatory and immunosuppressive, depending on the context and specific types of TILs present. Ideally, these immune cells penetrate tumors and dynamically modulate anti-tumor responses through direct cytotoxic activity, antigen presentation, and cytokine secretion. In this way, TILs play a fundamental role in enhancing anti-tumor immunity, and this beneficial effect is the main focus of this review. Indeed, TILs have gained considerable attention in cancer immunotherapy due to their potential to mediate tumor regression, making them a central focus in novel oncological treatments based on specific cell therapies (2). Their presence, functional activity, and spatial organization correlate with patient prognosis and therapeutic outcomes, particularly in immune checkpoint blockade (ICB) therapies (3).

TIL recruitment to tumors is primarily driven by chemokine signaling, where they interact with cancer cells and stromal components in a dynamic and often immunosuppressive environment (4). Despite their presence within tumors, many TILs exhibit functional exhaustion, which impairs their cytotoxic potential. This exhaustion is frequently driven by immune checkpoint molecule upregulation, metabolic competition, and the presence of inhibitory cytokines within the TME (5). The variability in TIL infiltration across different cancer types and individual patients has made them a critical subject of investigation in oncology research (6).

Recent advancements in TIL-based therapies have explored their adoptive transfer as a promising therapeutic strategy, particularly in melanoma, triple-negative breast cancer (TNBC), and colorectal cancer (7). Genetic and transcriptomic profiling has been instrumental in identifying TIL subpopulations that exhibit enhanced cytotoxic activity and persistence within the TME (8). These findings underscore the potential of TILs as both prognostic biomarkers and therapeutic agents in cancer immunotherapy.

While TIL-based therapies hold promise, effectively testing and evaluating TIL function in controlled environments remains a significant challenge. Preclinical *in vitro* and *in vivo* models have been developed to study TIL interactions with tumors and assess their therapeutic potential. Well-developed *in vitro* models such as 3D tumor spheroids and organoid cultures allow for examining TIL infiltration, persistence, and cytotoxicity in a controlled setting (9). These models enable researchers to manipulate immune and tumor interactions, facilitating the screening of novel immunotherapeutic agents. However, they often lack the complexity of an intact immune system and may not fully recapitulate the suppressive TME encountered *in vivo*.

*In vivo* preclinical models, including syngeneic mouse models, patient-derived xenografts (PDX), and humanized mouse models, provide a more comprehensive understanding of TIL behavior within a tumor-bearing organism (10). Syngeneic models involve implanting murine tumors into immunocompetent mice, preserving the native immune system and enabling TIL expansion and response to therapy (11). PDX models, in which patient-derived tumor cells are engrafted into immunocompromised mice, allow for studying human-specific TILs but lack a fully functional human immune system (12). Humanized mouse models have been developed to overcome this limitation, where human immune cells, including TILs, are introduced into immunodeficient mice to mimic human immune-tumor interactions (13). These models provide a critical platform for evaluating TIL-based therapies in a physiologically relevant setting, informing the development of clinical applications.

Despite these advances, there are inherent challenges in translating TIL research from preclinical models to clinical applications. Tumors exhibit significant heterogeneity in TIL infiltration, functional exhaustion, and immune evasion strategies, which can vary between *in vitro* and *in vivo* systems. Additionally, the immune system’s interactions with the TME remain highly complex, requiring the integration of multiple experimental models to accurately assess TIL function and therapeutic efficacy (14).

This review will comprehensively discuss the biological mechanisms driving TIL recruitment and activation within the TME, elucidating the key signaling pathways and cellular interactions that shape their function. Additionally, it will explore the prognostic and predictive value of TILs in cancer, highlighting their potential as biomarkers for patient stratification and response prediction in immunotherapy. The review will also address the major challenges and limitations associated with TIL-based therapies, including functional exhaustion, immune evasion mechanisms, and patient-specific variability. Finally, an in-depth analysis of preclinical models used for studying TIL-based immunotherapy will be presented, focusing on *in vitro* systems, animal models, and translational strategies to optimize TIL efficacy for clinical applications. This review aims to provide a foundation for future advancements in harnessing TILs for improved therapeutic outcomes by integrating insights from fundamental immunology and applied cancer research.

## 2 The cellular landscape of tumor-infiltrating lymphocytes: phenotypes, functions, and roles in anti-tumor immunity

The crucial role of the immune system in cancer surveillance and control has been recognized for over a century (15). Over the decades, extensive research has been devoted to understanding how immune cells detect, respond to, and eliminate malignant cells, leading to the development of immunotherapies aimed at restoring or enhancing anti-tumor immunity. Among the various immune cell populations, tumor-infiltrating lymphocytes have emerged as central orchestrators of the anti-cancer immune response. TILs are a heterogeneous group of lymphocytes—predominantly T cells—that infiltrate tumor tissues and exert both pro- and anti-tumor effects, depending on their phenotype, functional status, and interactions within the tumor microenvironment. Their recruitment is largely driven by chemokine gradients and inflammatory signals that guide their migration from peripheral blood into tumor sites.

The therapeutic efficacy and prognostic value of TILs are strongly influenced by their abundance, activation status, and spatial distribution within the tumor. These characteristics determine their ability to mount effective anti-tumor responses or contribute to immune evasion (16). Key subsets of TILs include CD8^+^ cytotoxic T lymphocytes (CTLs), CD4^+^ helper T cells, regulatory T cells (Tregs), B cells, and natural killer (NK) cells—each playing distinct roles in shaping tumor immunity.

Recent advances have also revealed that not only the quantity but the quality and metabolic fitness of TILs are critical for their anti-tumor functions (17). Single-cell RNA sequencing studies have uncovered profound heterogeneity within TIL populations, identifying specific transcriptional programs associated with persistence, stemness, and cytotoxic capacity (18). Moreover, the spatial localization of TILs relative to tumor cells, blood vessels, and stromal barriers has emerged as a major determinant of therapeutic responsiveness, with proximity to tumor islets correlating with better outcomes. New findings suggest that particular TIL subsets, such as stem-like progenitor exhausted T cells residing in tumor-draining lymph nodes or tertiary lymphoid structures, may be key drivers of durable responses to immunotherapy (19). Furthermore, modulation of the tumor microenvironment to enhance TIL metabolic fitness—such as promoting mitochondrial biogenesis and oxidative phosphorylation—represents a novel and promising strategy to boost TIL efficacy in solid tumors (20).

### 2.1 CD8+ cytotoxic T cells

CD8^+^ T cells are among the most prevalent effector cells within tumors, where they differentiate into CTLs upon antigen presentation by dendritic cells or other antigen-presenting cells (APCs). Once activated, CTLs release cytolytic granules containing perforin and granzymes, initiating apoptosis in tumor cells marked for destruction (21, 22). Perforin forms pores in the tumor cell membrane, allowing granzymes to enter and activate caspase-dependent and independent cell death pathways. Additionally, proteases such as cathepsins may amplify these cytotoxic effects (21).

Beyond direct killing, CTLs secrete cytokines like interferon-γ (IFNγ) and tumor necrosis factor-α (TNFα), which further stimulate anti-tumor immunity. Some CTLs transition into memory T cells, including tissue-resident memory T cells characterized by CD103 and CD39 expression, which have been associated with prolonged survival in various cancers (23–25). However, sustained antigen exposure can lead to T cell exhaustion, reducing their cytotoxic function and proliferative capacity. Nonetheless, a high density of CTLs, particularly within tertiary lymphoid structures (TLS), correlates with favorable prognosis in many tumor types (26), while the presence of memory subsets has been linked to reduced metastasis and improved disease-free survival (27, 28).

### 2.2 CD4+ helper T cells

CD4^+^ T cells constitute a major TIL subset and are critical for coordinating the adaptive immune response. Through the secretion of IFN-γ, TNF-α, and IL-2, these cells enhance CD8^+^ T cell cytotoxicity, promote Th1 polarization, and facilitate tumor antigen presentation. Upon activation by antigen presentation, naïve CD4^+^ T cells differentiate into effector subsets depending on cytokine cues and environmental context (29).

Among these, Th1 cells are essential in anti-tumor responses, driven by IL-12 and mediated via STAT signaling pathways, culminating in the expression of T-bet and the production of pro-inflammatory cytokines (29). These cytokines recruit and activate additional immune effectors, reinforcing local immunity. Tissue-resident memory CD4^+^ T cells have also shown promise as targets for immunotherapeutic intervention due to their robust, localized responses to tumor antigens (30).

### 2.3 Regulatory T cells

Tregs play a dual-edged role in the TME by preserving immune homeostasis while suppressing anti-tumor immunity. Identified by the expression of FOXP3, CD4^+^, CD25^+^, CTLA-4, and CD127^low^/–, Tregs limit immune activation through multiple mechanisms (31). They inhibit effector T cell function via PD-1 and CTLA-4, and secrete immunosuppressive cytokines like TGF-β, IL-10, and IL-35. Their high CD25 expression deprives surrounding effector T cells of IL-2, further limiting cytotoxic responses. Based on FOXP3 expression levels and other surface markers, the heterogeneity of Treg subsets suggests a nuanced regulatory function that could be therapeutically modulated to enhance anti-tumor responses (32).

### 2.4 B cells

B Cells infiltrated in the tumor were identified as good predictors of therapeutical response (33). These B cells can differentiate into plasma cells (effector B cells) to produce antibodies that target invading agents for destruction by macrophages or may become memory B cells. Memory B cells will help the immune system to elicit a faster response when encountering the same agent.

Recently, Ma et al. (34) examined tumor-infiltrating B cells across 21 different types of cancer and identified 15 subsets of tumor-associated B cells differentiated into antibody-secreting cells by either an extrafollicular pathway or by a germinal center pathway. Tumor types grouped into the extrafollicular pathway presented poor clinical outcomes and resistance to immunotherapy associated with glutamine-derived metabolites through epigenetic-metabolic cross-talk, which stimulated a T cell-driven immunosuppressive program. Ma et al. demonstrate the importance of the balance of intratumor B cell subsets and suggest that B cell–targeting immunotherapy could exploit humoral immunity.

### 2.5 Natural killer cells

NK cells, defined as CD56^+^CD3^-^ lymphocytes, are key players in innate anti-tumor immunity. They can eliminate tumor cells without prior sensitization by detecting stress-induced ligands and downregulated MHC class I molecules (35). NK cells mediate cytotoxicity through granzyme and perforin release and produce cytokines that modulate the immune landscape (36).

Subsets of NK cells—CD56^bright^ CD16^-^ and CD56^dim^ CD16^+^—exhibit distinct functional properties. While the latter is highly cytotoxic, the former can achieve potent cytolytic activity after IL-15 priming (37). NK cells also play a role in T cell recruitment and remodeling of the TME through cytokine release and death ligands like TRAIL and FASL (36). However, their function is often suppressed by Tregs, M2 macrophages, and inhibitory cytokines (e.g., IL-10, TGF-β), as well as checkpoint molecules like PD-1 and TIM-3, which contribute to early functional exhaustion (36, 38).

## 3 Regulation of TIL access and function within the tumor microenvironment

The infiltration, positioning, and functional activation of tumor-infiltrating lymphocytes within solid tumors are hallmarks of effective anti-tumor immunity. However, this process is highly complex and tightly regulated. The successful recruitment, entry, and activation of TILs are orchestrated by a multilayered network of molecular signals, structural components, and metabolic conditions that collectively determine whether immune cells can access tumor sites, survive within the hostile tumor microenvironment, and execute cytotoxic functions.

CD8^+^ cytotoxic T lymphocytes are the principal effectors in solid and hematologic malignancies, with their tumoricidal activity mediated by cytokines such as IFN-γ and TNF-α. However, persistent antigen exposure in the TME frequently induces T cell exhaustion—a dysfunctional state marked by upregulation of inhibitory receptors like PD-1 and CTLA-4 and diminished effector cytokine production. Immune checkpoint inhibitors targeting these pathways have shown significant success in reinvigorating TIL responses in solid tumors, while their application in hematologic malignancies remains less robust (26, 31, 39). Notably, the presence of tertiary lymphoid structures (TLS) within solid tumors correlates with enhanced TIL activation and improved clinical outcomes, as these ectopic lymphoid aggregates facilitate local antigen presentation and T cell priming (26). In contrast, hematological cancers are typically characterized by systemic immune activity and rarely form TLS, resulting in distinct immunological landscapes (**Table 1**).

**Table 1:** Comparison of TIL characteristics and behaviors in solid tumors versus hematological malignancies.

Feature	Solid Tumors	Hematological Malignancies	References
Types of TILs	CD8+ CTLs, CD4+ T helper cells, Tregs, B cells, NK cells	CD4+ T cells, CD8+ T cells, B cells, regulatory T cells	(40) (39) (41)
Functionality	Effector functions include cytotoxicity and cytokine release; often impaired by the TME	Generally maintain anti-tumor immunity; can be dysfunctionnal depending on the malignancy	(39) (41) (42)
Exhaustion Markers	High PD-1 and CTLA-4 expression linked to poor outcomes	PD-1 and CTLA-4 expression can also indicate exhaustion	(39)
TME Influence	Highly heterogeneous; immune suppression by Tregs and other immune cells prevalent	More uniform; micro-environment varies but generally supports immune responses	(40) (42) (43)
Prognostic Value	Higher TIL density is associated with better prognosis in many solid tumors (e.g., melanoma, breast cancer)	Variable; often context-dependent based on tumor type	(39) (41) (44)
Therapeutic Approaches	Adoptive cell therapy, checkpoint inhibitors (e.g., anti-PD-1) have shown promise.	CAR-T cell therapy is a major focus for treatment	(39) (42) (44)
Challenges	Tumor heterogeneity and immunosuppressive microenvironment limit the effectiveness of therapies	Resistance mechanisms can lead to treatment failure	(40) (41) (42)

This section explores the diverse biological systems that regulate TIL access and function—from initial chemotactic recruitment to physical entry across tumor vasculature and stromal barriers, and finally to their metabolic and immunologic engagement within the tumor core. Chemokines and cytokines establish navigational gradients for lymphocyte trafficking, influenced by inflammatory stimuli, oncogenic signaling, microbiota-derived factors, and tumor mutational burden. However, tumor-derived mechanisms such as decoy receptor expression, chemokine sequestration, or spatial mislocalization within stromal compartments can impede these gradients and undermine immune infiltration (45).

Structural components of the TME also impose significant physical and biochemical constraints. The tumor vasculature is frequently aberrant, leaky, and lacks the necessary adhesion molecules for efficient lymphocyte transmigration (46). Surrounding stromal elements—particularly cancer-associated fibroblasts (CAFs) and the extracellular matrix—further contribute to an immune-excluded phenotype by forming dense fibrotic barriers and secreting suppressive signals that limit immune cell penetration.

Even when TILs successfully infiltrate tumor tissues, their effector potential is threatened by a hostile microenvironment marked by nutrient deprivation, hypoxia, chronic antigen stimulation, and immunosuppressive cytokines. These factors promote T cell dysfunction and exhaustion, limiting sustained anti-tumor activity. Therefore, the transition from successful recruitment to effective cytotoxicity depends on a microenvironment that supports T cell metabolism, prevents exhaustion, and promotes immunological synapse formation (47).

This section provides an integrated analysis of the regulatory mechanisms that control TIL localization and function—spanning chemotaxis, stromal dynamics, vascular signaling, and immune activation. A deeper understanding of these processes is vital for the rational design of therapeutic strategies that not only guide TILs to tumors but also enable them to persist and function effectively within the TME.

### 3.1 Chemokine and cytokine networks governing TIL recruitment

The successful infiltration of tumor-infiltrating lymphocytes into tumors is a complex, highly regulated process controlled by networks of chemokines and cytokines. These soluble signaling molecules orchestrate immune cell trafficking by guiding T cells toward inflamed or malignant tissues via receptor-ligand interactions. Their expression, regulation, and spatial organization within the tumor microenvironment play a critical role in determining the quality and quantity of immune cell infiltration, directly impacting clinical outcomes and response to immunotherapy.

One of the most important chemokine-receptor pairs involved in TIL recruitment is the CXCL9/CXCL10/CXCL11–CXCR3 axis (48). These chemokines are potent chemo attractants for activated CD8+ and CD4+ Th1-type T cells that express the CXCR3 receptor. Studies have consistently shown that high levels of CXCL9 and CXCL10 in the TME are associated with greater CD8+ TIL density and improved survival in ovarian (49), breast (50), and colorectal cancers (51). For instance, in a study of advanced serous ovarian cancer, high expression of CXCL9 and CXCL10 predicted significantly better overall survival, and this was mechanistically linked to increased recruitment of CD8+ T cells via CXCR3 signaling (49).

Another key axis is CCL5–CCR5, which governs the trafficking of effector memory T cells (52). In renal cell carcinoma (RCC), tumor-infiltrating CD4+ T cells were found to predominantly express both CCR5 and CXCR3, supporting a Th1-polarized immune infiltrate. However, in metastatic RCC, there was a notable decrease in CCR5+ TILs and a rise in CCR4+ cells, suggesting a shift toward an immunosuppressive milieu during tumor progression (53).

Chemokine expression in tumors is not static—it is profoundly shaped by tumor-intrinsic factors such as oncogenic signaling and inflammatory cytokines. The IFN-γ signaling pathway, activated by T cells and NK cells, induces CXCL9 and CXCL10 expression in tumor and stromal cells (54). This creates a positive feedback loop that reinforces immune cell infiltration. Conversely, tumor cells can suppress this chemokine expression through activation of pathways like β-catenin, PI3K-AKT, or through overexpression of prostaglandin E2 (PGE2), which downregulates NF-κB-driven transcription of chemokines. COX inhibitors such as indomethacin were shown to restore CXCL9/10 expression in ovarian cancer models, while celecoxib suppressed it, indicating that even among COX inhibitors, the choice of agent can drastically alter immune infiltration outcomes (49).

The tumor’s mutational and microbial landscape also influences chemokine production. High tumor mutational burden (TMB) often correlates with elevated neoantigen load and IFN-γ production, leading to upregulation of CXCL9/10 and increased TIL recruitment. Moreover, in colorectal cancer, gut microbiota was shown to modulate chemokine expression directly. Bacterial components activated chemokine production (e.g., CXCL9, CXCL10, CCL5) by tumor cells, thereby enhancing T cell infiltration. Mice treated with antibiotics showed reduced chemokine levels and decreased TIL trafficking, highlighting a potential avenue for microbiota-based immunomodulation (55).

Finally, distinct subsets of chemokines also regulate the recruitment of other beneficial immune cells. CXCL13, for instance, is secreted by a specific transcriptionally distinct subset of CD103+CD8+ T cells under TGF-β signaling. This chemokine mediates B cell recruitment and tertiary lymphoid structure (TLS) formation in tumors, which is associated with enhanced anti-tumor immunity and checkpoint blockade responsiveness (56).

### 3.2 Endothelial adhesion molecules

Tumor-associated vasculature expresses adhesion molecules, such as ICAM-1 and VCAM-1, to facilitate lymphocyte transmigration. These molecules, belonging to the immunoglobulin superfamily of cell adhesion molecules (CAM), mediate the firm adherence of leukocytes to endothelial cells, a crucial step in leukocyte recruitment to inflammatory areas.

ICAM-1, an integrin ligand, is expressed on several malignant cells and may thus contribute to both cancer growth and cancer immunosurveillance by adaptive and non-adaptive immune arms (57). ICAM-1 regulates neutrophil adhesion and transcellular migration of TNF-α-activated vascular endothelium under flow (58).

While ICAM-1’s role in T cell crawling on initial lymphatics has been addressed, its specific role in tumor-infiltrating lymphocytes’ exit from tumors remains relatively unexplored (59). Blocking ICAM-1 in mice with intratumoral injections of activated T-lymphocytes led to significant increases in CD8+ T cell transit to the lymph nodes, suggesting that ICAM-1 blockage can decrease T-cell aggregates or clusters, with a parallel increment in oriented cell migration and transmigration across monolayers of lymphatic endothelial cells (59).

VCAM-1 mediates distinct tumor-stromal interactions that are unique to lung and bone microenvironments and facilitate metastasis to these sites when aberrantly expressed in breast cancer cells (60).

Monoclonal antibodies blocking ICAM-1 and VCAM-1 can efficiently inhibit DC adhesion and transmigration of dermal LEC monolayers *in vitro*, highlighting lymphatic transmigration as a potential new target for anti-inflammatory therapy. Transient local blockade of LFA-1/ICAM-1 functions offers an opportunity to attain systemic biodistribution of tumor-reactive T-lymphocytes (61). Elevated ICAM-1 expression in breast cancer cells results in a favorable outcome and prolonged survival of breast cancer patients (57). ICAM-1 expressed by metastatic breast cancer cells that expand inside the lung vasculature is involved in innate rather than in adaptive cancer cell killing, functioning as a suppressor of intravascular breast cancer metastasis to lungs (57). *Ex vivo*, neutrophils derived from tumor-bearing mice also killed cultured E0771 cells via ICAM-1-dependent interactions (57).

### 3.3 Dendritic cells

Dendritic cells (DCs) within the tumor microenvironment (TME) play a crucial role in antigen presentation, bridging innate and adaptive immune responses and priming naïve T cells for effector functions (62, 63). DCs are specialized antigen-presenting cells (APCs) that capture, process, and present tumor-associated antigens to T cells, initiating an adaptive immune response against the tumor (62, 63). DCs patrol the local environment, utilizing membrane and cytosolic receptors to recognize danger signals, including those from tumor cells (62, 63). Upon antigen uptake, DCs present these antigens to naïve T lymphocytes, initiating antigen-specific immune responses and regulating tolerance and immunity (62). DCs can present antigens via MHC class I and MHC class II molecules, activating CD8+ T cells and CD4+ T cells, respectively (64).

Different types of DCs exist within the TME, including conventional DCs (cDC1, cDC2, cDC3), monocyte-derived DCs (moDC), and plasmacytoid DCs (pDC), each with distinct roles (65). cDC1s are particularly important for cross-presentation, a process where they present antigens on MHC class I molecules to CD8+ T cells, leading to their activation and cytotoxic activity (63). A high percentage of cDC1s in the TME is generally associated with a better prognosis and favorable responses to immune checkpoint blockade (ICB) therapies (63). cDC2s, while less proficient in cross-presentation than cDC1s, effectively present MHC class II-related antigens to CD4+ T cells, promoting T helper cell responses (62, 63). The infiltration of CD4+ T cells in the TME has been correlated with the ratio of cDC2s to regulatory T cells (Tregs); a higher frequency of cDC2s correlates with greater CD4+ T-cell tumor infiltration (62).

The immunosuppressive TME impairs dendritic cell (DC) functions, inhibiting maturation, antigen presentation, and T cell activation, leading to immune tolerance and tumor progression (62, 63). Strategies to enhance antigen presentation and T cell priming are crucial for improving therapeutic outcomes (66). Novel approaches include DC vaccines pulsing DCs with tumor-associated antigens (66), reprogramming tumor cells into immunogenic cDC-like cells (67), and combining antigen presentation with other immunotherapies (66). The TME negatively regulates DC maturation, migration, and effector functions, with immunosuppressive populations like Tregs, MDSCs, and TAMs playing a significant role (68).

### 3.4 Functional polarization of TILs

Within the tumor microenvironment (TME), tumor-infiltrating lymphocytes (TILs) exhibit diverse functional polarizations, including effector T cells, exhausted T cells, and regulatory T cells (Tregs), each playing a significant, yet often opposing, role in anti-tumor immunity. Effector T cells, primarily CD8+ cytotoxic T lymphocytes (CTLs) and CD4+ helper T cells, are critical for directly targeting and eliminating tumor cells through the release of cytokines such as IFN-γ, TNF-α, and IL-2, and the use of cytotoxic granules containing perforin and granzymes (69). However, chronic antigen stimulation in the TME can lead to T cell exhaustion, characterized by the progressive loss of effector functions, reduced cytokine production, and diminished cytotoxicity. Exhausted T cells upregulate multiple inhibitory receptors (IRs), including PD-1, CTLA-4, TIM-3, LAG-3, and TIGIT, which bind to ligands on tumor cells and antigen-presenting cells (APCs), impeding T cell survival, expansion, and function (70). Furthermore, exhausted T cells exhibit diminished production of effector cytokines, such as IL-2, IFN-γ, and TNF-α, and have impaired cytotoxic activity (70).

The balance between effector T cell activity and suppression by Tregs is crucial in determining the overall immune response against the tumor. Tregs, a significant subset of TILs, actively suppress anti-tumor immunity through various mechanisms (71). These include the secretion of inhibitory cytokines such as IL-10 and TGF-β, which suppress the activity of effector T cells, NK cells, and DCs. TGF-β also induces the development of cancer-associated fibroblasts (CAFs), increasing extracellular matrix (ECM) production and deposition, thereby inhibiting effector T cell migration (71). Tregs express inhibitory receptors such as CTLA-4, PD-1, TIM-3, TIGIT, and LAG-3, with CTLA-4 inhibiting T cell activation by outcompeting CD28 for binding to B7 ligands on APCs. Tregs also disrupt T cell metabolism by expressing high levels of CD25 (IL-2 receptor), depriving surrounding effector T cells of IL-2, and by expressing ectonucleotidases CD39 and CD73, which convert ATP and ADP into adenosine, suppressing effector T cells (71). Given the opposing roles of effector T cells, exhausted T cells, and Tregs within the TME, therapeutic strategies aim to enhance effector T cell function while reversing exhaustion and suppressing Treg activity to improve cancer immunotherapy outcomes (70). Targeting molecules involved in Treg function, such as CTLA-4, can enhance anti-tumor immune responses, and combining checkpoint inhibitors with other therapies may further enhance anti-tumor immunity (70).

### 3.5 Stromal regulation of TIL entry

Effective infiltration of tumor-infiltrating lymphocytes into solid tumors is not solely determined by immune activation but is profoundly influenced by the tumor’s stromal architecture. The expression of endothelial adhesion molecules and the physical density and composition of the extracellular matrix (ECM)—primarily shaped by cancer-associated fibroblasts (CAFs)—constitute formidable barriers to TIL entry and distribution within the tumor parenchyma.

Adhesion molecules such as ICAM-1 (Intercellular Adhesion Molecule 1), VCAM-1 (Vascular Cell Adhesion Molecule 1), and E- and P-selectins are critical for leukocyte rolling, adhesion, and trans endothelial migration (72, 73). Under physiological conditions, these molecules are upregulated in response to inflammatory cytokines like TNF-α and IFN-γ (74).

Once T cells traverse the endothelium, they encounter the tumor stroma, a dense and fibrous environment composed of ECM components such as collagen, fibronectin, and hyaluronic acid. ECM remodeling, often driven by cancer-associated fibroblasts (CAFs), plays a dual role in both supporting tumor progression and regulating immune cell access (75). CAFs produce matrix metalloproteinases (MMPs) that modify the ECM and secrete chemokines that may either support or hinder TIL movement, depending on the subtype and inflammatory milieu. Moreover, they physically compartmentalize the tumor, creating immune exclusion zones where TILs accumulate at the invasive margins but fail to infiltrate the tumor core (76). This phenomenon is particularly characteristic of the immune-excluded phenotype, often observed in pancreatic and colorectal cancers.

An illustrative example comes from a study in breast cancer models, where tenascin-C, a matrix glycoprotein secreted by CAFs, was shown to trap CD8+ T cells in the stroma via its interaction with CXCL12. This stromal retention depended on TLR4 signaling and could be reversed by blocking the CXCL12-CXCR4 axis, restoring T cell migration into the tumor core and enhancing anti-tumor immunity (77). Such findings underscore the potential of stromal-targeted therapies to complement immune checkpoint inhibitors by facilitating T cell access.

### 3.6 Activation and effector function of TILs in the tumor microenvironment

The activation of tumor-infiltrating lymphocytes begins not within the tumor itself but in the tumor-draining lymph nodes (TDLNs), where naive T cells first encounter antigen-presenting cells (APCs) that have captured tumor antigens. This initiation process, known as T cell priming, is highly dependent on dendritic cell subsets, especially conventional type 1 dendritic cells (cDC1s), which specialize in the cross-presentation of tumor-derived antigens to CD8+ T cells (78). Recent studies have elucidated the central and multifaceted role that cDC1s play in orchestrating both CD8+ (79) and CD4+ (80) T cell responses, thereby determining the efficiency and durability of anti-tumor immunity. This dual capability enables cDC1s to serve as an independent platform for initiating T cell immunity while simultaneously coordinating the crucial crosstalk between helper and cytotoxic lymphocytes. CD4+ T cells, in turn, license cDC1s through CD40-CD40L interactions, enhancing their ability to activate CD8+ T cells, thus forming a tightly regulated feedback loop that amplifies the anti-tumor response (81).

The success of this priming process relies heavily on antigen availability and neoantigen quality. Tumors with high mutational burden tend to produce more neoantigens—novel peptides do not present in the normal host proteome—which are more likely to be recognized as foreign by the immune system. These high-quality neoantigens improve T cell priming efficacy and are associated with better responses to immunotherapy. However, tumor cells may evade detection by downregulating antigen presentation machinery or selecting for clones with lower immunogenicity, leading to immune escape. Moreover, as shown by Nayak et al. (82), the uptake of heat shock protein–chaperoned peptides by CD91+ cDC1s enables effective presentation of low-abundance tumor antigens, emphasizing the importance of antigen-chaperoning mechanisms during early tumor development (82).

However, the balance between immunogenic priming and tumor-induced tolerance is delicate. In certain anatomical locations, such as the pancreas or central nervous system, tumors may escape immune surveillance despite expressing recognizable neoantigens. This was highlighted by Diamond et al. (83), who found that pancreatic tumors with high antigenicity still failed to initiate effective CD8+ T cell responses due to poor cDC1 activation. This “site-dependent immune escape” could be reversed with CD40 agonists, restoring T cell priming and expanding the repertoire of tumor-reactive clones through epitope spreading (83).

Once primed in tumor-draining lymph nodes, tumor-infiltrating lymphocytes must sustain their activation, expand locally, and carry out cytotoxic functions within the immunosuppressive and metabolically hostile tumor microenvironment. It begins with the engagement of their TCRs with tumor-derived peptides presented on major histocompatibility complex (MHC) molecules, either by tumor cells directly or by intratumoral antigen-presenting cells (APCs). This recognition event triggers immunological synapse formation and initiates a cascade of downstream signaling involving phospholipase Cγ1 (PLCγ1), Ca²^+^ flux, calcineurin-NFAT activation, and ERK/MAPK pathways (84). These signals ultimately lead to transcriptional activation of genes responsible for cytokine production (e.g., IFN-γ, TNF-α), cytotoxic granule release (e.g., perforin, granzyme B), and clonal expansion.

**Figure 1:**
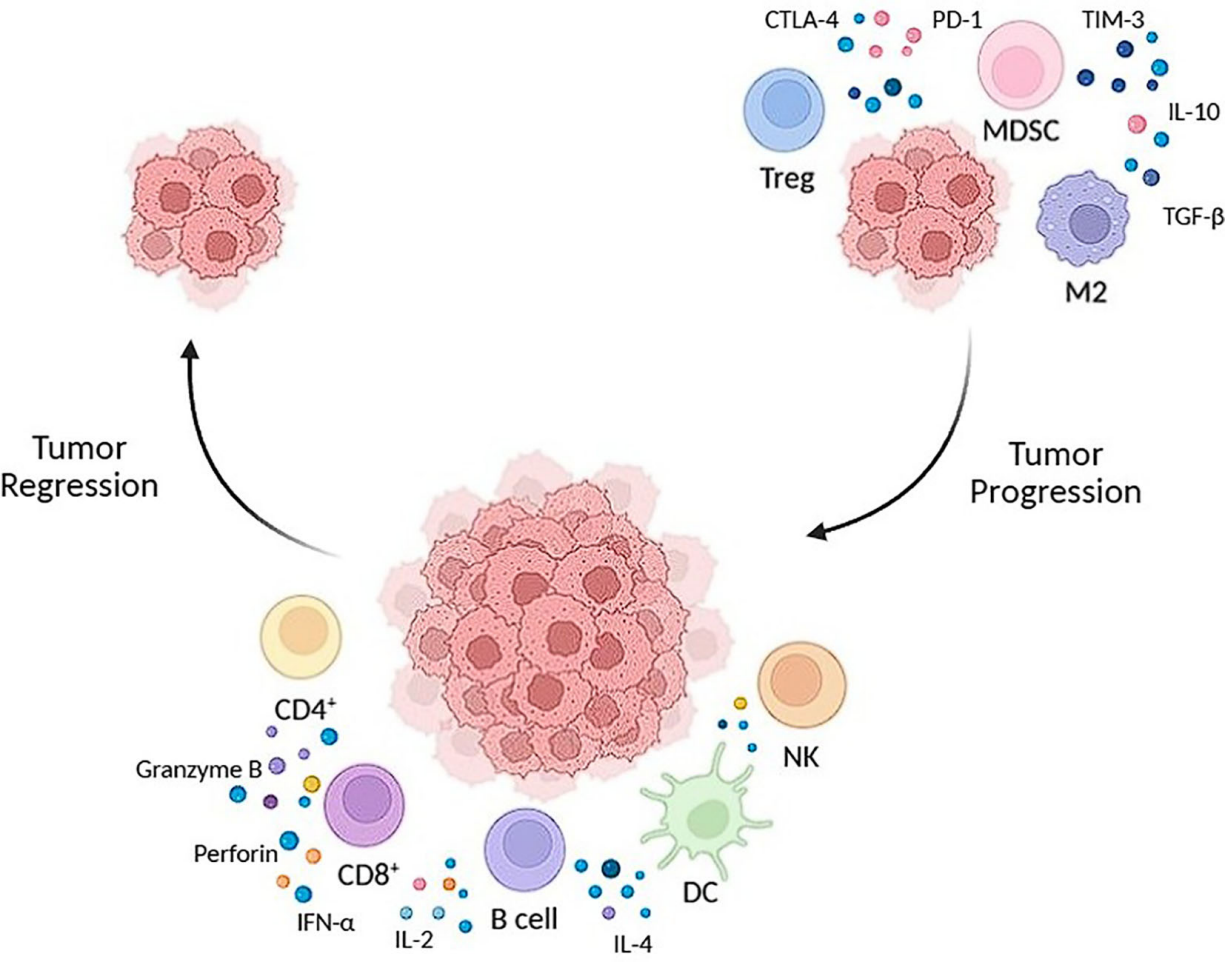
The summary of immune responses in tumor regression and progression. Tumor regression (left side) is driven by CD8, NK and dendritic cells, which release molecules to target the tumor cells. Tumor progression (right) is driven by an immunosuppressive environment caused by TREGS, MDSCS and M2, allowing tumor growth.

The summary of immune responses in tumor regression and progression can be seen on **Figure 1**.

## 4 Tumor-infiltrating lymphocytes as prognostic and predictive biomarkers

TILs have gained prominence as prognostic and predictive biomarkers across various cancers. Their presence, density, and composition in tumor tissues provide valuable insights into disease progression and therapeutic response. Standardized detection and quantification methods are crucial for validating TILs as reliable biomarkers across diverse populations. The importance of automated scoring methods in ensuring consistency and reproducibility in TIL assessments has been emphasized (85).

In breast cancer, TILs have demonstrated potential as significant biomarkers. However, heterogeneity in experimental designs and assessment methods has impeded a complete understanding of their prognostic value. The need for standardization in TIL evaluation is underscored by ongoing discussions regarding their biological and clinical significance (85). TILs have been extensively studied in HER2-positive breast cancer, with their presence correlating with various prognostic implications. A recent review consolidates findings on the prognostic significance of TILs in this subtype, suggesting their role in guiding therapeutic decisions. TIL assessment could be integrated into clinical practice to aid personalized treatment strategies and improve patient outcomes (86).

Efforts to establish reliable quantification methods for TILs led to introducing a standardized histological approach in 2014. This technique evaluates TIL percentages on hematoxylin and eosin (H&E)-stained slides, allowing for reproducibility across studies and reinforcing TILs as valid prognostic and predictive markers (87). While TILs are positively correlated with improved prognosis and chemotherapy response in triple-negative breast cancer (TNBC), their role in other breast cancer subtypes remains complex. Some subtypes show paradoxical associations between high TIL presence and poorer clinical outcomes, indicating the influence of tumor biology and immune interactions (88).

Despite TILs’ promising potential, inconsistencies in assessment methods and the need for standardized evaluation protocols hinder their clinical application. Further large-scale, well-controlled studies are essential to refine their role in oncological prognostication and integrate them into routine clinical practice. Standardized quantification techniques and additional immunological factor evaluations could enhance their utility in personalized treatment approaches (85, 89, 90).

Adoptive cell therapy (ACT) using TILs has demonstrated durable clinical responses in metastatic melanoma. This approach involves isolating, expanding, and reinfusing TILs to target cancer cells effectively. However, ACT remains complex, requiring extensive research to optimize patient selection and identify predictive biomarkers (91–94).

Studies have shown that specific lymphocyte subsets influence TIL therapy responses. Certain phenotypic characteristics of infused TILs are linked to clinical outcomes, highlighting the potential for TIL composition to serve as a predictive biomarker (91). Additionally, prior treatments, such as immune checkpoint inhibitors, affect TIL therapy efficacy, emphasizing the need for personalized treatment planning (95).

Peripheral immune biomarkers have also been associated with TIL therapy responses. Research identifying biomarkers in peripheral blood suggests potential predictive tools for assessing treatment success (96). Comprehensive biomarker research is crucial to refining patient selection criteria and improving TIL therapy outcomes in metastatic melanoma.

TILs play a vital role as prognostic and predictive biomarkers in solid tumors. Their density, presence, and immune composition are significant indicators of tumor behavior and patient outcomes (97, 98).

### 4.1 Prognostic significance of TILs

TILs are associated with better survival in several cancers, including breast cancer, melanoma, and non-small cell lung cancer (NSCLC) (99–102). A high density of CD8+ cytotoxic T lymphocytes within tumors is particularly linked to favorable outcomes, reflecting an active immune response against cancer cells (97, 103). Recent studies further highlight the prognostic role of different TIL subsets in specific cancer types. For instance, higher intratumoral CD4+ and stromal CD8+ counts in breast cancer were independently associated with improved survival, suggesting their potential as prognostic biomarkers (104). Similarly, in triple-negative breast cancer (TNBC), higher levels of TILs were correlated with prolonged overall survival and disease-free survival (105).

TILs are a strong prognostic factor in colorectal cancer, particularly in stage III disease, where a high TIL density was associated with significantly better disease-free survival (106). Similarly, in ovarian cancer, the presence and degree of TIL infiltration were significantly linked to patient survival. They could be a key factor in identifying patients who might benefit from immunotherapy.

Additionally, in NSCLC, a meta-analysis of 60 studies found that patients with higher TIL infiltration had significantly improved overall survival, particularly among CD8+, CD3+, and CD4+ subtypes (102). These findings emphasize the importance of TIL density and phenotype as independent prognostic markers across various malignancies, reinforcing their role in shaping the tumor immune microenvironment and influencing patient outcomes.

Although high TIL density is often linked to better outcomes in some cancers (97, 107), this isn’t always consistent across all cases. TIL prognostic value varies with their phenotype, function, and spatial context (108, 109). Without considering factors like T cell exhaustion or the presence of immunosuppressive cells such as Tregs, simply measuring TIL levels may lead to misleading conclusions (110).

### 4.2 Predictive value of TILs

TIL presence in tumors is increasingly recognized as a predictor of response to immunotherapies, especially immune checkpoint inhibitors like anti-PD-1/PD-L1 therapies. Tumors with robust CD8+ T cell infiltration are more likely to respond positively to these treatments (111, 112). Dynamic interactions between TILs and tumor cells also influence chemotherapy and targeted therapy responses, impacting tumor progression and patient outcomes (113, 114).

In breast cancer, high TIL levels have been shown to predict response to neoadjuvant chemotherapy, particularly in triple-negative and HER2-positive subtypes. Patients with high TIL densities often experience better pathological complete response (pCR) rates, indicating their role in guiding treatment decisions (86, 87). Additionally, in hormone receptor-positive breast cancer, the predictive value of TILs is less pronounced, suggesting the need for additional biomarkers to refine therapeutic strategies (88).

TIL composition and functionality play a crucial role in predicting response to immune checkpoint inhibitors for melanoma. Studies have demonstrated that tumors enriched with activated CD8+ T cells exhibit better responses to anti-PD-1 and anti-CTLA-4 therapies, supporting their predictive utility (98, 115). Moreover, TIL phenotypic markers, such as PD-1 and LAG-3 expression, have been explored as indicators of exhaustion and therapeutic response (116).

In lung cancer, the predictive value of TILs is increasingly recognized, particularly in NSCLC. High levels of CD8+ T cells and their spatial distribution within the tumor microenvironment are associated with enhanced responses to immunotherapies. PD-L1 expression in conjunction with TIL levels has been used to stratify patients likely to benefit from immune checkpoint inhibitors (100, 102).

Colorectal cancer patients with high TIL densities, especially those with a TH1-polarized immune profile, have demonstrated superior responses to immunotherapies. Microsatellite instability-high (MSI-H) tumors, characterized by abundant TILs, show significant sensitivity to checkpoint blockade therapies, reinforcing the predictive role of TILs in guiding immunotherapy choices (97, 114).

Overall, TILs are valuable predictive biomarkers across multiple cancer types, guiding treatment selection and improving patient outcomes. Further refinement of TIL assessment methodologies and integration with additional immune markers could enhance their clinical utility in precision oncology.

## 5 Limitations and resistance mechanisms of TIL therapy

TILs, as effectors of the adaptive immune system, can recognize and destroy malignant cells through their antigen-specific cytotoxic responses. These lymphocytes originate from the host’s immune repertoire. They are recruited into the tumor microenvironment, where they can directly kill tumor cells, produce cytokines such as IFN-γ and TNF-α, and promote broader anti-tumor immunity (117). The presence of TILs, particularly CD8+ cytotoxic T cells and certain subsets of CD4+ T helper cells, within the TME is widely recognized as a favorable prognostic marker across multiple solid tumors, including melanoma, non-small cell lung cancer, bladder cancer, breast cancer, and ovarian cancer (118). This strong correlation with improved clinical outcomes provides the rationale for adoptive cell therapy using TILs, which involves isolating and expanding tumor-reactive lymphocytes from patient tumor samples and reinfusing them after lymphodepletion. TIL therapy has demonstrated promising results in melanoma, achieving durable responses in some patients resistant to other forms of immunotherapy (95). However, this therapeutic strategy remains limited by both intrinsic and extrinsic barriers that diminish TIL efficacy *in vivo* (119).

Intrinsic factors include tumor heterogeneity, loss of neoantigen expression, and TIL exhaustion due to chronic antigen stimulation. Extrinsic barriers are shaped by the immunosuppressive TME, characterized by regulatory cells (e.g., Tregs, MDSCs, M2 macrophages), inhibitory cytokines (TGF-β, IL-10), metabolic stressors, and checkpoint ligand expression (e.g., PD-L1, VISTA). Moreover, poor tumor antigenicity in low-mutational burden cancers impairs initial T cell priming and recruitment. TILs may also fail to infiltrate tumors adequately due to physical barriers in the stroma or a lack of appropriate chemokine signals (120).

Despite ongoing efforts to optimize cell expansion, selection of tumor-reactive clones, and combination with immune checkpoint inhibitors or other modulatory agents, many patients still experience relapse or do not respond to TIL therapy at all. These failures highlight the need to better understand and therapeutically modulate the complex interplay between TILs and the TME (121). Current research is focusing on improving TIL persistence, overcoming exhaustion, and enhancing tumor infiltration through genetic engineering and combination strategies.

### 5.1 Tumor microenvironment phenotypes and immunological landscapes

The immunological characteristics of the TME are broadly classified into three phenotypes: inflamed, immune-excluded, and immune-desert. These phenotypes predict differential responses to immunotherapy and shape TIL activity (120).

The inflamed TME is typified by abundant infiltration of CD8+ cytotoxic T lymphocytes (CTLs), CD4+ helper T cells, NK cells, and antigen-presenting cells. These immune cells mediate anti-tumor activity, but their function is often impaired by immunosuppressive populations such as regulatory T cells (Tregs), myeloid-derived suppressor cells (MDSCs), and tumor-associated macrophages (TAMs) (119). These cells upregulate inhibitory ligands and secrete immunosuppressive cytokines, contributing to T cell exhaustion (122).

CD4+ T cell subsets, particularly Th2 and Th17, contribute to tumor progression by promoting TAM and MDSC recruitment via IL-4, IL-13, and IL-17-driven pathways (123, 124). However, Th17 cells also display dual roles—exerting anti-tumor effects through IFN-γ and chemokine-mediated recruitment of effector immune cells (123). Tregs are particularly abundant in the inflamed TME, suppressing CTL and NK cell activity via surface-bound and secreting TGF-β and IL-10 (125, 126). Their accumulation strongly correlates with poor prognosis and resistance to immune checkpoint inhibitors (ICIs) (127).

In the immune-excluded phenotype, immune cells are retained in the peritumoral stroma, unable to infiltrate tumor nests due to physical barriers like dense collagen matrices and an unfavorable chemokine milieu (128, 129). This exclusion hampers effective T cell-tumor cell interaction and renders tumors less responsive to TIL and ICI therapies.

Immune-Desert TME is characterized by the paucity or complete absence of TILs within both the tumor and surrounding stroma. Tumors in this category often exhibit low mutational burden and neoantigen expression, resulting in impaired T cell priming and immunological ignorance (130, 131). Deficiencies in antigen presentation—through HLA I downregulation or β2-microglobulin mutations—further exacerbate immune evasion (132). The immune-desert TME also harbors immunosuppressive cell types like TAMs, Tregs, and MDSCs inhibiting dendritic cell (DC) maturation and activation (133).

### 5.2 Key immunosuppressive cell populations

The tumor microenvironment is heavily infiltrated by immunosuppressive cells that collectively inhibit the activation, expansion, and cytotoxic function of tumor-infiltrating lymphocytes. These cells—especially tumor-associated macrophages (TAMs), myeloid-derived suppressor cells (MDSCs), and regulatory T cells (Tregs)—orchestrate a suppressive network that interferes with anti-tumor immunity on multiple levels, contributing significantly to resistance against TIL therapy and immune checkpoint inhibitors.

TAMs are among the most abundant immune cells within the TME and exhibit high plasticity, capable of polarizing into two main functional states: classically activated (M1) and alternatively activated (M2) macrophages. M1 macrophages play a pro-inflammatory, anti-tumoral role. They are typically induced by IFN-γ, TNF-α, and microbial products like LPS, and express high levels of inducible nitric oxide synthase (iNOS), reactive oxygen species (ROS), and IL-12. M1 macrophages promote tumor destruction by directly killing tumor cells and by enhancing the recruitment and activation of cytotoxic CD8+ T cells and natural killer (NK) cells via secretion of CXCL9, CXCL10, and CXCL11 chemokines (134). They also secrete TNF-α and IL-1β, which amplify T cell responses and facilitate antigen presentation.

In contrast, M2 macrophages are stimulated by IL-4, IL-10, IL-13, and glucocorticoids and exhibit a strongly immunosuppressive, pro-tumoral phenotype. They express arginase-1, CD206, and secrete high levels of IL-10 and TGF-β, both suppressing T cell responses. M2 TAMs promote tumor progression by expressing PD-L1, remodeling the extracellular matrix through matrix metalloproteinases (MMP2, MMP9), enhancing angiogenesis via vascular endothelial growth factor (VEGF), and recruiting immunosuppressive cells such as Tregs. They facilitate epithelial-to-mesenchymal transition (EMT) and metastasis through cytokines such as CCL18 and TGF-β (135). Moreover, the ratio of M1 to M2 macrophages within tumors is increasingly recognized as a prognostic indicator: high M2 infiltration is correlated with poor outcomes in many cancers, including breast, lung, and colorectal cancer (136).

MDSCs are a heterogeneous population of immature myeloid cells that expand during cancer, inflammation, and infection. Tumors are differentiated into two main subtypes: monocytic (M-MDSCs) and polymorphonuclear or granulocytic (PMN-MDSCs). MDSCs are potent suppressors of both innate and adaptive immunity. They inhibit T cell receptor signaling and effector function through multiple mechanisms, including expression of arginase-1 (ARG1), inducible nitric oxide synthase (iNOS), and the production of ROS and reactive nitrogen species (RNS) (137). These mechanisms collectively deplete L-arginine, nitrate tyrosine residues on TCR complexes, and downregulate CD3ζ chain expression, thereby silencing T cell activation.

MDSCs also impair NK cell cytotoxicity by downregulating activating receptors such as NKG2D, and suppress DC maturation, leading to inefficient antigen presentation. Significantly, MDSCs facilitate the expansion and recruitment of Tregs by producing IL-10, TGF-β, and by expressing membrane-bound TGF-β (mTGF-β), further dampening the anti-tumor immune response (138, 139). Tumor-derived inflammatory cytokines, including IL-6, IL-1β, IL-8, GM-CSF, and VEGF, support the expansion, survival, and migration of MDSCs to the tumor site, establishing a chronic state of immune suppression (140). Elevated MDSC levels in the peripheral blood and tumors of cancer patients have been associated with poor prognosis and reduced response to immunotherapy.

Tregs, primarily characterized by CD4+CD25+Foxp3+ expression, are central to maintaining immune homeostasis and self-tolerance under physiological conditions. However, in the tumor setting, their expansion is co-opted to suppress anti-tumor immunity. Tregs accumulate in large numbers within the TME and exert their suppressive effects via multiple pathways. They secrete immunosuppressive cytokines such as TGF-β and IL-10, directly inhibit the proliferation and cytotoxic activity of CD8+ T cells and NK cells and suppress the maturation and antigen-presenting capacity of dendritic cells (125).

Treg stability and function are supported by IL-10 and insulin-like growth factors (IGFs), which also promote the expansion of MDSCs and the immunosuppressive M2 macrophage phenotype. These molecular interactions create a feedback loop within the TME that maintains a state of immune privilege for the tumor (141–143). High Treg infiltration is consistently associated with poor clinical outcomes, especially in cancers such as ovarian, pancreatic, and hepatocellular carcinoma.

Moreover, Tregs express high levels of immune checkpoint receptors like CTLA-4, PD-1, TIM-3, LAG-3, and TIGIT, and they can outcompete effector T cells for IL-2, thereby promoting exhaustion and anergy in TILs (144). Through CTLA-4-mediated downregulation of CD80/CD86 on antigen-presenting cells and the delivery of suppressive signals via contact-dependent mechanisms, Tregs function as key mediators of immune evasion. In addition to immune suppression, Tregs contribute to tumor angiogenesis by secreting VEGF and enhancing M2 macrophage polarization.

### 5.3 Metabolic challenges in the TME

The tumor microenvironment imposes unique and profound metabolic constraints on tumor-infiltrating lymphocytes, significantly impairing their effector functions and persistence. One of the hallmark features of solid tumors is hypoxia, resulting from the rapid proliferation of cancer cells outpacing their blood supply. Hypoxic conditions disrupt oxidative phosphorylation in TILs and lead to the stabilization of hypoxia-inducible factors (HIFs), particularly HIF-1α, which alters T cell metabolism toward a less efficient glycolytic phenotype. While effector T cells also rely on glycolysis, the simultaneous nutrient depletion within the TME severely restricts this adaptation (145).

Rapidly dividing tumor cells consume glucose and essential amino acids such as glutamine, arginine, and tryptophan at a much higher rate than surrounding immune cells, creating a state of nutrient scarcity. This competition limits the availability of key metabolic substrates required for TIL proliferation, activation, and cytokine production. For instance, glucose deprivation impairs glycolytic flux and reduces IFN-γ production, a key cytokine in anti-tumor immunity (146). Similarly, arginine deprivation, often mediated by the enzyme arginase secreted by myeloid-derived suppressor cells (MDSCs), blocks T cell proliferation and reduces CD3ζ expression, which is essential for TCR signaling (147).

Amino acid catabolism is another major mechanism by which tumors create an immunosuppressive metabolic niche. Indoleamine-2,3-dioxygenase (IDO) and tryptophan 2,3-dioxygenase (TDO), both upregulated in many tumors and dendritic cells within the TME, degrade tryptophan into kynurenine. Elevated levels of kynurenine suppress T cell function by inducing T cell anergy, promoting regulatory T cell differentiation, and activating aryl hydrocarbon receptor (AhR)-mediated immunoregulatory pathways. The depletion of tryptophan itself inhibits mTOR signaling, essential for T cell metabolism and activation (69).

Furthermore, lactic acid, a byproduct of anaerobic glycolysis heavily employed by tumor cells (the Warburg effect), accumulates in the TME and acidifies the extracellular environment. Acidification inhibits T cell motility, survival, and their ability to form immunological synapses with tumor cells. It also suppresses cytotoxic activity and cytokine secretion by effector CD8+ T cells. High lactate levels have been associated with reduced infiltration and function of TILs and are now considered a barrier to successful immunotherapy (148).

Recent studies also highlight how mitochondrial dysfunction in TILs—caused by oxidative stress, mitochondrial DNA damage, and impaired biogenesis—contributes to their functional exhaustion. The energy-depleted, ROS-rich environment within tumors further promotes the expression of inhibitory receptors such as PD-1, TIM-3, and LAG-3, reinforcing the exhausted phenotype of T cells and diminishing their capacity to persist and eliminate tumor cells (149).

To address these challenges, new strategies are being explored, including metabolic reprogramming of TILs ex vivo, use of metabolic adjuvants like metformin to enhance mitochondrial function, and inhibition of enzymes such as IDO or arginase. These approaches aim to restore metabolic fitness and effector capacity of TILs and improve the clinical efficacy of adoptive T cell therapies and checkpoint inhibitors.

### 5.4 Manufacturing and expansion limitations

Another major limitation in TIL therapy is the ex vivo expansion process. Traditional rapid expansion protocols (REPs) using feeder cells and high-dose IL-2 often lead to TIL exhaustion and reduced *in vivo* persistence (150). Advanced platforms now aim to address these drawbacks. For instance, CRISPR/Cas9-engineered TIL products like KSQ-001EX knock out negative regulators such as SOCS1, enhancing TIL sensitivity to cytokines and promoting cytotoxic function (151). In preclinical models, these engineered cells retain a diverse TCR repertoire and show potent anti-tumor activity.

Similarly, the GT316 TIL product, generated by dual knockout of GT304 and GT312 via CRISPR/Cas9, exhibited robust tumor control *in vivo* with reduced dependence on IL-2 (152). These innovations in TIL manufacturing represent critical steps toward improving clinical scalability and durability of responses.

The effectiveness of TIL therapy is fundamentally shaped by the immunosuppressive forces of the TME, epigenetic and metabolic barriers, and limitations in manufacturing and cell persistence. Innovations in gene editing, metabolic reprogramming, and biomarker-guided personalization are paving the way to enhance TIL therapy across a broader range of solid tumors. Future therapeutic success will likely depend on integrating TIL therapy with combination strategies that target multiple axes of resistance, from checkpoint inhibition and cytokine modulation to targeting suppressive stromal and myeloid cell populations.

## 6 From bench to bedside: experimental models guiding the development of next-generation TIL immunotherapies

The rapid evolution of tumor-infiltrating lymphocyte-based immunotherapies demands sophisticated experimental models that faithfully recapitulate the complex interplay between tumor cells, the immune microenvironment, and therapeutic interventions. As the clinical relevance of TIL therapy expands beyond melanoma into diverse solid tumors, there is a pressing need for robust and translationally relevant platforms to investigate the biological mechanisms governing TIL recruitment, activation, persistence, and therapeutic efficacy.

A fundamental challenge in TIL research lies in bridging the gap between the highly controlled, reductionist nature of *in vitro* studies and the intricate, system-wide dynamics observed in human tumors. To meet this challenge, researchers have developed a spectrum of model systems—ranging from simple two-dimensional (2D) cultures to advanced three-dimensional (3D) organoid-TIL co-cultures and from immunocompetent murine syngeneic models to humanized mouse systems capable of supporting human immune-tumor interactions. Each platform offers unique advantages and limitations and collectively serves as the foundation for preclinical development, functional validation, and optimization of next-generation TIL therapies.

*In vitro* and *in vivo* experimental models form the backbone of TIL-based immunotherapy research, each offering distinct advantages for understanding and optimizing TIL behavior and therapeutic efficacy. *In vitro* systems—ranging from traditional 2D cytotoxicity assays to more advanced 3D tumor spheroids, patient-derived organoids, and microfluidic or bioprinted devices—enable controlled, high-throughput analysis of TIL-tumor interactions. These models allow researchers to dissect mechanisms of cytotoxicity, immune evasion, chemokine responsiveness, and drug synergy in a tractable setting. They are particularly valuable for testing gene edits, evaluating cytokine dependencies, and profiling functional responses across various tumor types.

In contrast, *in vivo* models offer a more comprehensive view of TIL dynamics in a physiologically relevant environment. Syngeneic mouse models, which preserve immune-competent settings, remain foundational for assessing murine TIL infiltration, expansion, memory formation, and therapeutic efficacy. Humanized mouse models further enable the study of gene-engineered human TILs and their activity against patient-derived xenografts (PDXs), providing a critical bridge toward clinical application.

This chapter synthesizes the latest developments in both *in vitro* and *in vivo* platforms used to investigate TIL function, engineering, and translational potential. Detailing the design, utility, and limitations of these systems highlights how preclinical modeling informs the rational development of next generation TIL therapies and accelerates their progression from bench to bedside in cancer immunotherapy.

### 6.1 Advanced *in vitro* platforms for modeling TIL–tumor interactions and optimizing immunotherapy

*In vitro* models have become essential platforms for studying tumor-infiltrating lymphocyte (TIL)–tumor interactions under controlled and reproducible conditions. 2D co-culture systems remain foundational for rapid, high-throughput TIL-mediated cytotoxicity and activation assessments. However, they lack the spatial and biochemical context of *in vivo* tumors. To address these limitations, 3D tumor spheroids have gained traction. These models recapitulate important features of tumor architecture, such as proliferation gradients, hypoxic cores, and stromal barriers. Recent studies have shown that 3D spheroids significantly enhance TIL activation, expansion, and cytotoxicity compared to 2D systems, especially when combined with immune checkpoint blockade like PD-1 inhibition (153).

Patient-derived organoid (PDO) systems co-cultured with autologous TILs offer even greater translational relevance. Platforms like those described by Liu et al. allow tracking of real-time infiltration and tumor-specific killing in autologous settings, while also enabling immune-phenotypic analysis and drug response profiling (154).

Researchers, such as the EVIDENT platform, have developed microfluidic systems that incorporate dynamic perfusion, oxygen gradients, and immune cell flow to improve physiological fidelity. This device allows for real-time imaging of autologous TIL-tumor fragment interactions and the assessment of immune checkpoint inhibitor efficacy (155).

Bioprinted models also present new opportunities for recapitulating spatial features of the tumor microenvironment (TME) (156). Flores-Torres et al. employed a multicomponent hydrogel co-culture tumor-immune model that simulates TIL migration and functional activation (157). Other studies using laser-based bioprinting offer precise control over spheroid size and geometry to fine-tune drug response assays (158).

### 6.2 Tumor-level preclinical models

Murine tumor models remain the cornerstone for evaluating the *in vivo* functionality of engineered tumor-infiltrating lymphocytes, enabling researchers to assess T cell expansion, trafficking, persistence, tumor infiltration, and therapeutic efficacy in an intact immunological environment. Among these, syngeneic tumor models, in which murine tumors are implanted into immunocompetent mice of the same genetic background, offer a robust system for dissecting immune-tumor interactions and testing next-generation TIL products before clinical translation.

The B16-OVA melanoma model is one of the most widely used systems for evaluating antigen-specific TILs. It expresses the model antigen ovalbumin (OVA), which allows for precise tracking of TCR-specific responses (e.g., OT-I CD8+ T cells) (159). This model is instrumental in testing variables such as lymphodepletion regimens, cytokine support (e.g., IL-2, IL-15), and routes of TIL administration (e.g., intravenous vs. intratumoral).

A landmark study by Wong et al. (160) utilized the B16-OVA model to evaluate dual-edited TILs with CRISPR-mediated knockout of Regnase-1 and SOCS1. These two transcriptional repressors act as intracellular immune checkpoints (160). The dual knockout resulted in over 3,500-fold increased TIL infiltration, robust IFN-γ production, and complete tumor eradication, surpassing the performance of single-edited TILs. The engineered TILs exhibited improved survival, polyfunctionality, and metabolic fitness within the TME, confirming that simultaneous targeting of multiple negative regulators could dramatically enhance therapeutic potency (160).

Beyond B16 melanoma, other models such as MC38 colon carcinoma (161), and 4T1 breast cancer (162) provide platforms for evaluating TIL therapy in more immunosuppressive or immune-excluded environments. These models help researchers assess the impact of stromal barriers, tumor antigen heterogeneity, and spatial localization of TILs. Notably, in orthotopic models—where tumors grow in their tissue of origin—TIL trafficking and local immune suppression more accurately reflect clinical settings, allowing for better prediction of therapeutic success.

Recent innovations have also introduced humanized mouse models, where immunodeficient mice are engrafted with human tumors and immune cells. These models facilitate the study of human TILs *in vivo* and allow direct testing of gene-edited human TIL products (e.g., PD-1 KO or synthetic TCR-TILs) (163). Such systems have been essential for optimizing TIL expansion protocols and validating neoantigen-specific responses prior to initiating early-phase clinical trials.

Moreover, tumor rechallenge experiments in murine models are used to test the formation of T cell memory. Mice that rejected tumors after adoptive TIL therapy are re-injected with tumor cells weeks or months later to assess whether long-term immunological protection has been established (164).

In conclusion, tumor-level preclinical models—particularly syngeneic and humanized murine systems—are indispensable tools for advancing TIL therapy. They enable precise functional dissection of engineered T cells, facilitate biomarker discovery, and accelerate the translation of next generation TIL products from bench to bedside.

## 7 Therapeutic strategies to enhance TIL recruitment and activation

The therapeutic potential of tumor-infiltrating lymphocyte-based immunotherapy depends not only on the intrinsic quality and tumor-reactivity of the infused lymphocytes but also on the receptiveness of the tumor microenvironment to support their infiltration, activation, and persistence. Given the numerous immune barriers posed by solid tumors—ranging from immunosuppressive cytokines and metabolic stress to stromal exclusion and checkpoint inhibition—multiple complementary strategies have been developed to potentiate TIL function. These include immune checkpoint inhibitors, costimulatory agonists, innate immune activators, chemokine modulation, and strategies to normalize tumor vasculature.

One of the foundational pillars of TIL-enhancing strategies is immune checkpoint blockade. Monoclonal antibodies targeting PD-1, CTLA-4, and LAG-3 relieve inhibitory signals that contribute to T cell exhaustion and functional anergy within the TME (165). These therapies have revolutionized treatment for several cancers by reinvigorating endogenous and adoptively transferred T cells. Recent innovations have expanded this paradigm to include intracellular checkpoints such as Cytokine-Inducible SH2-containing protein (CISH), a suppressor of TCR signaling. Deletion of CISH using CRISPR/Cas9 technology has been shown to enhance TIL sensitivity to tumor neoantigens, boost cytokine secretion (e.g., IFN-γ), and improve responses to PD-1 blockade in preclinical models, laying the groundwork for combinatorial strategies that target both surface and intracellular checkpoints (166).

In parallel, costimulatory receptor agonists—such as anti-4-1BB (CD137) and anti-OX40—are being investigated to further enhance TIL expansion and survival following activation (167). These molecules augment IL-2 production and promote the development of long-lived effector and memory T cells, thus improving TIL persistence in hostile tumor environments. The synergistic potential of combining checkpoint inhibitors with costimulatory agonists is currently being evaluated in clinical trials, with early-phase studies showing enhanced T cell proliferation and improved tumor control (168).

Beyond adaptive immune modulation, innate immune agonists are gaining traction as tools to reshape the immunological landscape of tumors and facilitate TIL infiltration. STING (Stimulator of Interferon Genes) agonists, such as ADU-S100, activate cytosolic DNA sensing pathways that drive the production of type I interferons and chemokines including CXCL10 and VEGI. Intratumoral administration of STING agonists has been shown to normalize tumor vasculature, recruit dendritic cells, and promote the formation of tertiary lymphoid structures (TLS)—niches that support local T cell priming and expansion (169). Notably, endogenous STING signaling upregulates CXCL10 and CCL5 in mismatch repair-deficient colorectal cancers, facilitating dense CD8+ T cell infiltration. These findings highlight the therapeutic promise of exogenous STING activation in otherwise poorly immunogenic tumors (170).

Additional innate immune strategies include oncolytic viruses and toll-like receptor (TLR) ligands, which stimulate pattern recognition receptors (PRRs) on tumor and immune cells, leading to enhanced antigen presentation and immune cell recruitment (171, 172). These agents increase the visibility of tumor cells to the immune system and create inflammatory conditions favorable for TIL expansion and effector function. Together, these emerging approaches illustrate a multi-pronged therapeutic arsenal aimed at unlocking the full potential of TIL therapy by transforming immune-cold tumors into immune-active sites primed for T cell–mediated destruction.

## 8 Technology driven insights and emerging directions

**Figure 2:**
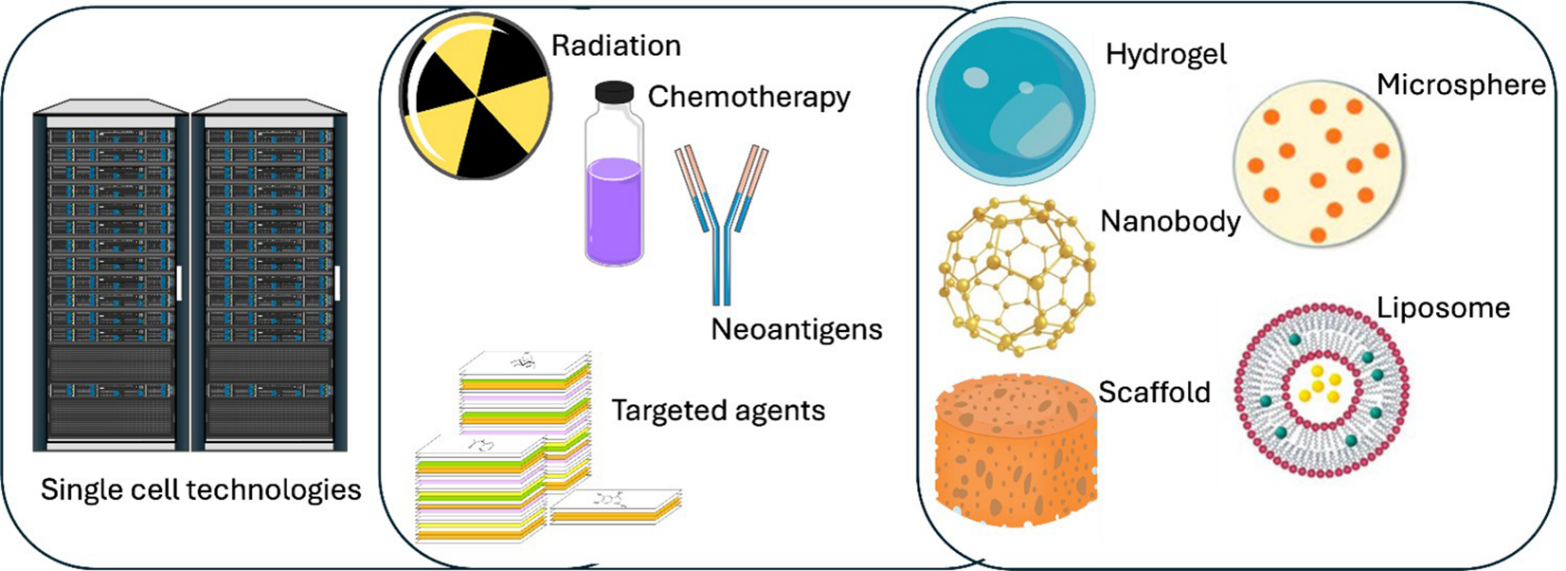
Overview of a multifaceted cancer treatment paradigm integrating single cell technologies with diverse therapeutic strategies. High-performance computing (left) is used to process large-scale single cell data, along the identification of patient-specific neoantigens and the selection of targeted agents. Conventional therapies, including radiation and chemotherapy, are combined with advanced modalities such as various biomaterials (right). By uniting data-driven insights with both established and emerging therapies, such a framework may optimize and personalize TIL-based cancer treatment for improved patient outcomes.

Molecular and computational biology advances continuously transform our understanding of tumor-infiltrating lymphocytes (TILs) and their application in cancer immunotherapy. Despite significant strides in current therapies, emerging research avenues promise to refine further and enhance the efficacy of TIL-based approaches. This section reviews several key innovations - including single-cell sequencing, neoantigen targeting, combination therapies, and biomaterial strategies - and discusses how these technologies may address current challenges in TIL research and pave the way for next-generation therapies (**Figure 2**).

Single-cell RNA sequencing (scRNA-seq) has emerged as a transformative tool in immuno-oncology by allowing researchers to profile individual cells within the tumor microenvironment (TME). This technology has several critical advantages (18). It enables the identification of diverse TIL subpopulations that may differ in activation status, exhaustion profiles, or cytotoxic potential (1). By cataloging these differences, researchers can pinpoint which subsets are most effective at mediating anti-tumor responses. Beyond phenotypic classification, scRNA-seq also provides insights into the dynamic functional states of TILs, including cytokine production, metabolic activity, and engagement of key signaling pathways. These insights are crucial for understanding the mechanisms underlying T cell exhaustion and resistance to immunotherapy. Furthermore, the high-resolution data generated by single-cell approaches can inform the development of predictive biomarkers, aiding in selecting and expanding the most therapeutically potent TIL subsets for adoptive cell therapy.

Emerging protocols now combine scRNA-seq with spatial transcriptomics, enabling researchers to map the spatial distribution of TILs in relation to other cells in the TME (173). These advances enhance our understanding of immune cell dynamics and inform the design of interventions to selectively enrich for beneficial TIL populations.

Next, neoantigen targeting and combination therapies represent a transformative avenue for enhancing TIL specificity through personalized immunotherapies. Neoantigens, tumor-specific antigens arising from somatic mutations, can activate TILs with high specificity against cancer cells. Advances in computational biology have identified patient-specific neoantigens, facilitating the design of personalized vaccines or adoptive T-cell therapies. Huber et al. recently reported NeoDisc, an advanced computational framework designed to identify and predict clinically relevant antigenic peptides on cancer cells. For this, genomic, transcriptomic and immunopeptidomic data are integrated to accurately identify peptides originating from tumor specific antigens, mutations, oncoviral elements or noncanonical sources. Hence, a personalized proteome reference is generated for each individual and their tumor lesions, including annotating such tumor-specific alterations (174). These approaches aim to improve the efficacy of TIL-based interventions while minimizing off-target effects. Of note, integrating TIL-based therapies with other treatment modalities has shown promise in amplifying therapeutic efficacy. Combination strategies involving chemotherapy, radiotherapy, immune checkpoint inhibitors, and targeted agents can synergistically enhance TIL recruitment, activation, and persistence within tumors. For instance, immune checkpoint blockade can alleviate T-cell exhaustion by targeting inhibitory pathways such as PD-1/PD-L1 or CTLA-4, thereby boosting the anti-tumor activity of TILs. Similarly, radiotherapy has been shown to modulate the TME by increasing antigen presentation and chemokine production, fostering a more favorable environment for TIL infiltration.

More than ever, biomaterials represent a promising frontier in cancer immunotherapy, providing innovative solutions to overcome the physical and biochemical barriers that limit TIL infiltration and activity within solid tumors (175). These materials have been shown to incorporate ECM-driven cues to influence immune cells, fitting into the 3D architecture and altering or tuning immune cell phenotypes. A rich set of materials can be engineered to achieve biocompatibility and deliver immunomodulatory agents, such as cytokines, chemokines, or small molecules, directly to the tumor site, creating a localized immune-stimulatory environment. Key examples include nanoparticle delivery systems, hydrogels and scaffolds designed to mimic extracellular matrix components that serve as supportive niches for TIL expansion and persistence within the TME. The TME is characterized by hypoxia, acidity, high interstitial pressure, and a dense extracellular matrix, all hindering TIL infiltration and activity. Biomaterials can be engineered to specifically target these barriers, helping to normalize the TME and improve immune cell function. For instance, nanoparticles designed to deliver oxygen or neutralize acidic conditions can alleviate hypoxia and acidity within tumors, thereby enhancing TIL activation and cytotoxicity (176). Similarly, biomaterials that disrupt the dense ECM can facilitate TIL penetration into deeper tumor regions, improving their access to malignant cells. Hydrogels infused with CCL21 or CXCL9 have increased lymphocyte migration into tumors. These biomaterial systems enhance recruitment and sustain localized immune activation by providing a controlled release of immunomodulatory agents.

One of the most promising applications of biomaterials is their ability to reshape the immunosuppressive TME by neutralizing inhibitory signals or enhancing antigen presentation. For example, nanoparticles loaded with immune checkpoint inhibitors or stimulatory cytokines can selectively target tumor-associated immune cells, reinvigorating exhausted TILs and promoting robust anti-tumor responses. Additionally, biomaterials can recruit TILs by delivering chemokines that attract lymphocytes to the tumor site. Recent studies have demonstrated the potential of biomaterials in combination therapies (177). By integrating biomaterial-based approaches with other modalities, such as chemotherapy or radiotherapy, researchers aim to enhance TIL recruitment and activation synergistically. Biomaterial scaffolds combined with radiotherapy have increased antigen presentation and fostered a more favorable environment for TIL infiltration (178). These advancements underscore the versatility of biomaterials in addressing multiple challenges associated with TIL-based immunotherapy. Interestingly, Inambar et al. reported a cell-free polymer implant designed to recruit, genetically reprogram and expand host T cells at tumor lesions *in situ* (178) (**Table 2**).

**Table 2:** Advancing TIL-based therapies.

Emerging approach	Key advances	Benefits	Considerations
Single-Cell Technologies	High-resolution profiling of individual TILs to identify distinct subpopulations, functional states, and cellular interactions.	Enables the identification of TILs with enhanced cytotoxic activity and persistence.	Careful analysis required to avoid inadvertently promoting immunosuppressive subsets or disrupting beneficial immune-stromal interactions.
	Integration with spatial transcriptomics.	Can guide the development of targeted therapies to reinvigorate exhausted TILs.	High data complexity. Need for robust computational tools to analyze and interpret data
	Allows understanding of TIL heterogeneity and exhaustion mechanisms.	Enhanced biomarker discovery and patient stratification	
Neoantigen Targeting	Personalized immunotherapies that target tumor-specific antigens arising from somatic mutations.	Enhances TIL specificity and minimizes off-target effects, leading to more effective tumor cell killing.	Requires thorough validation to ensure neoantigen selection drives robust and specific anti-tumor immunity, avoiding tolerance or immune evasion.
	Utilizes computational biology to identify patient-specific neoantigens.	Increased TIL specificity for tumor cells	Accurate prediction of immunogenic neoantigens
	Next-generation sequencing and bioinformatic prediction	Personalized immunotherapies tailored to individual tumor profiles	Rapid, patient-specific manufacturing required
	Customized TIL expansion protocols		
Combination Therapies	Integration of TIL-based therapies with other treatment modalities (chemotherapy, radiotherapy, targeted agents, immune checkpoint inhibitors)	Synergistically enhances TIL recruitment, activation, and persistence within tumors.	Requires careful sequencing and dosing to maximize synergistic effects while minimizing toxicity and the potential for promoting tumor growth or metastasis.
		Can overcome immune suppression, resistance mechanisms and improve overall efficacy.	
Biomaterial Innovations	Engineering materials (scaffolds, hydrogels, nanoparticles) to deliver immunomodulatory agents directly to the tumor site, modulate the TME, and enhance TIL recruitment, expansion, and function.	Improves TIL infiltration, overcomes physical and biochemical barriers, and enhances localized immune activation within the TME.	Precise control over biomaterial properties and release kinetics is essential to avoid off-target effects, excessive inflammation, or unintended promotion of tumor progression.
	Can normalize TME, deliver cytokines, or serve as supportive niches.		

In summary, the convergence of single-cell technologies, neoantigen targeting, combinatorial treatment regimens, and biomaterial innovations is paving the way for the next generation of TIL-based therapies. These emerging strategies hold significant promise for refining personalized immunotherapy approaches and improving outcomes for cancer patients. Of course, reshaping the TME shall occur with care to minimize the risk of tumor growth and distant metastasis.

## 9 Conclusions

Tumor-infiltrating lymphocytes (TILs) have emerged as a cornerstone of modern cancer immunotherapy, offering the unique advantage of harnessing the patient’s own tumor-specific immune repertoire to target and eliminate malignant cells. Their natural infiltration into tumors and broad antigen recognition capabilities position TILs as powerful agents for personalized adoptive cell therapy. Clinical successes, particularly in metastatic melanoma, have demonstrated their capacity to induce durable responses—even in patients refractory to checkpoint inhibitors—thereby validating the therapeutic potential of TILs in solid tumors (179, 180).

Despite these promising outcomes, TIL therapy faces a complex array of biological and technical challenges that limit its broader application. One of the primary barriers lies within the tumor microenvironment (TME), a highly immunosuppressive landscape that impedes T cell infiltration, activation, and persistence. Factors such as stromal exclusion, vascular dysfunction, metabolic deprivation, and immune checkpoint engagement collectively contribute to TIL dysfunction and exhaustion. Regulatory immune cells, including Tregs, myeloid-derived suppressor cells (MDSCs), and M2-polarized tumor-associated macrophages (TAMs), further shape this suppressive ecosystem by producing inhibitory cytokines (e.g., TGF-β, IL-10) and depleting key metabolic substrates required for TIL fitness (119, 181).

Additionally, the intrinsic heterogeneity of solid tumors—ranging from antigenic diversity to variable mutational burden—affects the immunogenicity and composition of the TIL pool. In poorly immunogenic or “immune-cold” tumors, the absence of chemokine gradients and dendritic cell priming mechanisms leads to suboptimal TIL recruitment and ineffective priming in tumor-draining lymph nodes (78, 83). In such cases, therapeutic strategies that restore chemokine signaling (e.g., CXCL9/10-CXCR3 axis), enhance antigen presentation, or normalize tumor vasculature are essential to reinvigorate TIL activity within the TME (48, 169).

To overcome these limitations, researchers have developed a suite of innovative approaches aimed at boosting TIL recruitment, effector function, and persistence. These include the use of immune checkpoint inhibitors (e.g., anti-PD-1, anti-CTLA-4), costimulatory agonists (e.g., 4-1BB, OX40), and innate immune activators such as STING agonists and oncolytic viruses. Genetic engineering of TILs via CRISPR/Cas9 to delete inhibitory regulators like CISH or SOCS1 has shown promise in preclinical models, leading to enhanced tumor infiltration, polyfunctionality, and complete tumor regression (160, 166).

A parallel frontier in TIL therapy advancement is the development of sophisticated experimental models that replicate the complexity of human tumors and guide therapeutic optimization. *In vitro* systems, including 3D tumor spheroids, patient-derived organoids (PDOs), and microfluidic tumor-on-a-chip devices, enable precise dissection of TIL-tumor interactions under controlled conditions. These platforms facilitate high-throughput testing of cytokine dependencies, gene edits, and drug combinations in autologous contexts (154, 157). Meanwhile, *in vivo* models—particularly syngeneic and humanized mouse models—offer essential insights into TIL trafficking, memory formation, and therapeutic efficacy in an immunocompetent setting. Humanized models further enable testing of engineered TIL products against patient-derived xenografts, bridging the gap between preclinical validation and clinical translation (163).

As manufacturing platforms continue to evolve, rapid expansion protocols and GMP-compliant workflows are refined to generate potent TIL products at a clinical scale. New-generation products such as IOV-4001, GT316, and KSQ-001EX reflect a shift toward genetically enhanced, IL-2-independent, and functionally robust TILs suitable for broad clinical deployment (182–184). Moreover, novel *in situ* TIL therapies—such as local mRNA delivery of activating ligands—may further streamline treatment by obviating ex vivo manipulation.

In conclusion, TIL-based immunotherapy represents a rapidly maturing field that harnesses the adaptive immune system’s natural specificity and potency to combat cancer. While challenges related to tumor heterogeneity, immune suppression, and TIL exhaustion remain, advances in model systems, combination strategies, and genetic engineering are accelerating the field’s trajectory toward broader and more effective clinical application. Integrating biomarker-driven patient selection, improved manufacturing, and rationally designed combinatorial regimens is key to unlocking the full therapeutic potential of TILs in solid tumors.

## References

[1] Zhang H Chen L Li L Liu Y Das B Zhai S. Prediction and analysis of tumor infiltrating lymphocytes across 28 cancers by TILScout using deep learning. NPJ Precis Oncol. (2025) 9:76. doi: 10.1038/s41698-025-00866-0 40108446 PMC11923303

[2] Kumar A Watkins R Vilgelm AE. Cell therapy with TILs: training and taming T cells to fight cancer. Front Immunol. (2021) 12:690499. doi: 10.3389/fimmu.2021.690499 34140957 PMC8204054

[3] Zito Marino F Ascierto PA Rossi G Staibano S Montella M Russo D. Are tumor-infiltrating lymphocytes protagonists or background actors in patient selection for cancer immunotherapy? Expert Opin Biol Ther. (2017) 17:735–46. doi: 10.1080/14712598.2017.1309387 28318336

[4] Yi M Li T Niu M Zhang H Wu Y Wu K. Targeting cytokine and chemokine signaling pathways for cancer therapy. Signal Transduct Target Ther. (2024) 9:176. doi: 10.1038/s41392-024-01868-3 39034318 PMC11275440

[5] Jenkins E Whitehead T Fellermeyer M Davis SJ Sharma S. The current state and future of T-cell exhaustion research. Oxf Open Immunol. (2023) 4:iqad006. doi: 10.1093/oxfimm/iqad006 37554723 PMC10352049

[6] Brummel K Eerkens AL De Bruyn M Nijman HW. Tumour-infiltrating lymphocytes: from prognosis to treatment selection. Br J Cancer. (2023) 128:451–8. doi: 10.1038/s41416-022-02119-4 PMC9938191 36564565

[7] Matsueda S Chen L Li H Yao H Yu F. Recent clinical researches and technological development in TIL therapy. Cancer Immunol Immunother. (2024) 73:232. doi: 10.1007/s00262-024-03793-4 39264449 PMC11393248

[8] Zeng Z Zhang T Zhang J Li S Connor S Zhang B. A minimal gene set characterizes TIL specific for diverse tumor antigens across different cancer types. Nat Commun. (2025) 16:1070. doi: 10.1038/s41467-024-55059-3 39900903 PMC11791090

[9] Mu P Zhou S Lv T Xia F Shen L Wan J. Newly developed 3D *in vitro* models to study tumor-immune interaction. J Exp Clin Cancer Res. (2023) 42:81. doi: 10.1186/s13046-023-02653-w 37016422 PMC10074642

[10] Bareham B Georgakopoulos N Matas-Cespedes A Curran M Saeb-Parsy K. Modeling human tumor-immune environments *in vivo* for the preclinical assessment of immunotherapies. Cancer Immunol Immunother. (2021) 70:2737–50. doi: 10.1007/s00262-021-02897-5 PMC8423639 33830275

[11] Carretta M Thorseth ML SChina A Agardy DA Johansen AZ Baker KJ. Dissecting tumor microenvironment heterogeneity in syngeneic mouse models: insights on cancer-associated fibroblast phenotypes shaped by infiltrating T cells. Front Immunol. (2023) 14:1320614. doi: 10.3389/fimmu.2023.1320614 38259467 PMC10800379

[12] Liu Y Wu W Cai C Zhang H Shen H Han Y. Patient-derived xenograft models in cancer therapy: technologies and applications. Signal Transduct Target Ther. (2023) 8:160. doi: 10.1038/s41392-023-01419-2 37045827 PMC10097874

[13] Brehm MA Shultz LD Luban J Greiner DL. Overcoming current limitations in humanized mouse research. J Infect Dis. (2013) 208 Suppl 2:S125–130. doi: 10.1093/infdis/jit319 PMC3807974 24151318

[14] Tiwari A Trivedi R Lin SY. Tumor microenvironment: barrier or opportunity towards effective cancer therapy. J BioMed Sci. (2022) 29:83. doi: 10.1186/s12929-022-00866-3 36253762 PMC9575280

[15] Curti BD. Checkpoint immunotherapy for melanoma - offering hope for cure. N Engl J Med. (2025) 392:81–2. doi: 10.1056/NEJMe2412226 39752303

[16] Paijens ST Vledder A De Bruyn M Nijman HW. Tumor-infiltrating lymphocytes in the immunotherapy era. Cell Mol Immunol. (2021) 18:842–59. doi: 10.1038/s41423-020-00565-9 PMC8115290 33139907

[17] Siska PJ Rathmell JC. T cell metabolic fitness in antitumor immunity. Trends Immunol. (2015) 36:257–64. doi: 10.1016/j.it.2015.02.007 PMC4393792 25773310

[18] Andreatta M Corria-Osorio J Müller S Cubas R Coukos G Carmona SJ. Interpretation of T cell states from single-cell transcriptomics data using reference atlases. Nat Commun. (2021) 12:2965. doi: 10.1038/s41467-021-23324-4 34017005 PMC8137700

[19] Jiang W He Y He W Wu G Zhou X Sheng Q. Exhausted CD8+T cells in the tumor immune microenvironment: new pathways to therapy. Front Immunol. (2021) 11. doi: 10.3389/fimmu.2020.622509 PMC7902023 33633741

[20] Wang J He Y Hu F Hu C Sun Y Yang K. Metabolic reprogramming of immune cells in the tumor microenvironment. Int J Mol Sci. (2024) 25:12223. doi: 10.3390/ijms252212223 39596288 PMC11594648

[21] Chowdhury D Lieberman J. Death by a thousand cuts: granzyme pathways of programmed cell death. Annu Rev Immunol. (2008) 26:389–420. doi: 10.1146/annurev.immunol.26.021607.090404 18304003 PMC2790083

[22] Hay ZLZ Slansky JE. Granzymes: the molecular executors of immune-mediated cytotoxicity. Int J Mol Sci. (2022) 23. doi: 10.3390/ijms23031833 PMC8836949 35163755

[23] Corgnac S Boutet M Kfoury M Naltet C Mami-Chouaib F. The emerging role of CD8(+) tissue resident memory T (T(RM)) cells in antitumor immunity: A unique functional contribution of the CD103 integrin. Front Immunol. (2018) 9:1904. doi: 10.3389/fimmu.2018.01904 30158938 PMC6104123

[24] Duhen T Duhen R Montler R Moses J Moudgil T De Miranda NF. Co-expression of CD39 and CD103 identifies tumor-reactive CD8 T cells in human solid tumors. Nat Commun. (2018) 9:2724. doi: 10.1038/s41467-018-05072-0 30006565 PMC6045647

[25] Kim Y Shin Y Kang GH. Prognostic significance of CD103+ immune cells in solid tumor: a systemic review and meta-analysis. Sci Rep. (2019) 9:3808. doi: 10.1038/s41598-019-40527-4 30846807 PMC6405906

[26] Melssen MM Sheybani ND Leick KM Slingluff CL. Barriers to immune cell infiltration in tumors. J ImmunoTherapy Cancer. (2023) 11:e006401. doi: 10.1136/jitc-2022-006401 PMC10124321 37072352

[27] Klebanoff CA Gattinoni L Torabi-Parizi P Kerstann K Cardones AR Finkelstein SE. Central memory self/tumor-reactive CD8+ T cells confer superior antitumor immunity compared with effector memory T cells. Proc Natl Acad Sci U S A. (2005) 102:9571–6. doi: 10.1073/pnas.0503726102 PMC1172264 15980149

[28] Ahmadvand S Faghih Z Montazer M Safaei A Mokhtari M Jafari P. Importance of CD45RO+ tumor-infiltrating lymphocytes in post-operative survival of breast cancer patients. Cell Oncol (Dordr). (2019) 42:343–56. doi: 10.1007/s13402-019-00430-6 PMC12994353 30825183

[29] Bevington SL Cauchy P Withers DR Lane PJ Cockerill PN. T cell receptor and cytokine signaling can function at different stages to establish and maintain transcriptional memory and enable T helper cell differentiation. Front Immunol. (2017) 8:204. doi: 10.3389/fimmu.2017.00204 28316598 PMC5334638

[30] Montauti E Oh DY Fong L. CD4(+) T cells in antitumor immunity. Trends Cancer. (2024) 10:969–85. doi: 10.1016/j.trecan.2024.07.009 PMC11464182 39242276

[31] Piroozkhah M Gholinezhad Y Piroozkhah M Shams E Nazemalhosseini-Mojarad E. The molecular mechanism of actions and clinical utilities of tumor infiltrating lymphocytes in gastrointestinal cancers: a comprehensive review and future prospects toward personalized medicine. Front Immunol. (2023) 14:1298891. doi: 10.3389/fimmu.2023.1298891 38077386 PMC10704251

[32] Tay C Tanaka A Sakaguchi S. Tumor-infiltrating regulatory T&xa0;cells as targets of cancer immunotherapy. Cancer Cell. (2023) 41:450–65. doi: 10.1016/j.ccell.2023.02.014 36917950

[33] Laumont CM Banville AC Gilardi M Hollern DP Nelson BH. Tumour-infiltrating B cells: immunological mechanisms, clinical impact and therapeutic opportunities. Nat Rev Cancer. (2022) 22:414–30. doi: 10.1038/s41568-022-00466-1 PMC9678336 35393541

[34] Ma J Wu Y Ma L Yang X Zhang T Song G. A blueprint for tumor-infiltrating B cells across human cancers. Science. (2024) 384:eadj4857. doi: 10.1126/science.adj4857 38696569

[35] Wolf NK Kissiov DU Raulet DH. Roles of natural killer cells in immunity to cancer, and applications to immunotherapy. Nat Rev Immunol. (2023) 23:90–105. doi: 10.1038/s41577-022-00732-1 35637393

[36] Coënon L Geindreau M Ghiringhelli F Villalba M Bruchard M. Natural Killer cells at the frontline in the fight against cancer. Cell Death Dis. (2024) 15:614. doi: 10.1038/s41419-024-06976-0 39179536 PMC11343846

[37] Wagner JA Rosario M Romee R Berrien-Elliott MM Schneider SE Leong JW. CD56bright NK cells exhibit potent antitumor responses following IL-15 priming. J Clin Invest. (2017) 127:4042–58. doi: 10.1172/JCI90387 PMC5663359 28972539

[38] Dean I Lee CYC Tuong ZK Li Z Tibbitt CA Willis C. Rapid functional impairment of natural killer cells following tumor entry limits anti-tumor immunity. Nat Commun. (2024) 15:683. doi: 10.1038/s41467-024-44789-z 38267402 PMC10808449

[39] Kazemi MH Sadri M Najafi A Rahimi A Baghernejadan Z Khorramdelazad H. Tumor-infiltrating lymphocytes for treatment of solid tumors: It takes two to tango? Front Immunol. (2022) 13. doi: 10.3389/fimmu.2022.1018962 PMC9651159 36389779

[40] Hendry S Salgado R Gevaert T Russell PA John T Thapa B. Assessing tumor-infiltrating lymphocytes in solid tumors: A practical review for pathologists and proposal for a standardized method from the international immuno-oncology biomarkers working group: part 2: TILs in melanoma, gastrointestinal tract carcinomas, non-small cell lung carcinoma and mesothelioma, endometrial and ovarian carcinomas, squamous cell carcinoma of the head and neck, genitourinary carcinomas, and primary brain tumors. Adv Anat Pathol. (2017) 24:311–35. doi: 10.1097/PAP.0000000000000161 PMC5638696 28777143

[41] Buisseret L Soizic G Alexandre DW Gert VDE Anais B Cinzia S. Tumor-infiltrating lymphocyte composition, organization and PD-1/PD-L1 expression are linked in breast cancer. OncoImmunology. (2017) 6:e1257452. doi: 10.1080/2162402X.2016.1257452 28197375 PMC5283629

[42] Li B. Why do tumor-infiltrating lymphocytes have variable efficacy in the treatment of solid tumors? Front Immunol. (2022) 13. doi: 10.3389/fimmu.2022.973881 PMC9635507 36341370

[43] Stanton SE Disis ML. Clinical significance of tumor-infiltrating lymphocytes in breast cancer. J ImmunoTherapy Cancer. (2016) 4:59. doi: 10.1186/s40425-016-0165-6 PMC5067916 27777769

[44] Ogiya R Niikura N Kumaki N Bianchini G Kitano S Iwamoto T. Comparison of tumor-infiltrating lymphocytes between primary and metastatic tumors in breast cancer patients. Cancer Sci. (2016) 107:1730–5. doi: 10.1111/cas.2016.107.issue-12 PMC5198965 27727484

[45] Safaei S Yari A Pourbagherian O Maleki LA. The role of cytokines in shaping the future of Cancer immunotherapy. Cytokine. (2025) 189:156888. doi: 10.1016/j.cyto.2025.156888 40010034

[46] Cavaco A Rezaei M Niland S Eble JA. Collateral damage intended-cancer-associated fibroblasts and vasculature are potential targets in cancer therapy. Int J Mol Sci. (2017) 18. doi: 10.3390/ijms18112355 PMC5713324 29112161

[47] Mun S Lee HJ Kim P. Rebuilding the microenvironment of primary tumors in humans: a focus on stroma. Exp Mol Med. (2024) 56:527–48. doi: 10.1038/s12276-024-01191-5 PMC10984944 38443595

[48] Tokunaga R Zhang W Naseem M Puccini A Berger MD Soni S. CXCL9, CXCL10, CXCL11/CXCR3 axis for immune activation - A target for novel cancer therapy. Cancer Treat Rev. (2018) 63:40–7. doi: 10.1016/j.ctrv.2017.11.007 PMC5801162 29207310

[49] Bronger H Singer J Windmuller C Reuning U Zech D Delbridge C. CXCL9 and CXCL10 predict survival and are regulated by cyclooxygenase inhibition in advanced serous ovarian cancer. Br J Cancer. (2016) 115:553–63. doi: 10.1038/bjc.2016.172 PMC4997538 27490802

[50] Kim M Choi HY Woo JW Chung YR Park SY. Role of CXCL10 in the progression of *in situ* to invasive carcinoma of the breast. Sci Rep. (2021) 11:18007. doi: 10.1038/s41598-021-97390-5 34504204 PMC8429587

[51] Li W Li K Chen Y Wang S Xu K Ye S. IRF1 transcriptionally up-regulates CXCL10 which increases CD8(+) T cells infiltration in colorectal cancer. Int Immunopharmacol. (2025) 144:113678. doi: 10.1016/j.intimp.2024.113678 39591825

[52] Ozga AJ Chow MT Luster AD. Chemokines and the immune response to cancer. Immunity. (2021) 54:859–74. doi: 10.1016/j.immuni.2021.01.012 PMC8434759 33838745

[53] Cozar JM Canton J Tallada M Concha A Cabrera T Garrido F. Analysis of NK cells and chemokine receptors in tumor infiltrating CD4 T lymphocytes in human renal carcinomas. Cancer Immunol Immunother. (2005) 54:858–66. doi: 10.1007/s00262-004-0646-1 PMC11032824 15887015

[54] Liu M Guo S Stiles JK. The emerging role of CXCL10 in cancer (Review). Oncol Lett. (2011) 2:583–9. doi: 10.3892/ol.2011.300 PMC3406435 22848232

[55] Cremonesi E Governa V Garzon JFG Mele V Amicarella F Muraro MG. Gut microbiota modulate T cell trafficking into human colorectal cancer. Gut. (2018) 67:1984–94. doi: 10.1136/gutjnl-2016-313498 29437871

[56] Workel HH Lubbers JM Arnold R Prins TM van der Vlies P De Lange K. A transcriptionally distinct CXCL13(+)CD103(+)CD8(+) T-cell population is associated with B-cell recruitment and neoantigen load in human cancer. Cancer Immunol Res. (2019) 7:784–96. doi: 10.1158/2326-6066.CIR-18-0517 30872264

[57] Regev O Kizner M Roncato F Dadiani M Saini M Castro-Giner F. ICAM-1 on breast cancer cells suppresses lung metastasis but is dispensable for tumor growth and killing by cytotoxic T cells. Front Immunol. (2022) 13. doi: 10.3389/fimmu.2022.849701 PMC9328178 35911772

[58] Yang L Froio RM Sciuto TE Dvorak AM Alon R Luscinskas FW. ICAM-1 regulates neutrophil adhesion and transcellular migration of TNF-α-activated vascular endothelium under flow. Blood. (2005) 106:584–92. doi: 10.1182/blood-2004-12-4942 PMC1635241 15811956

[59] Yanguas A Garasa S Teijeira Á Aubá C Melero I Rouzaut A. ICAM-1-LFA-1 dependent CD8+ T-lymphocyte aggregation in tumor tissue prevents recirculation to draining lymph nodes. Front Immunol. (2018) 9. doi: 10.3389/fimmu.2018.02084 PMC6143661 30258446

[60] Chen Q Massagué J. Molecular pathways: VCAM-1 as a potential therapeutic target in metastasis. Clin Cancer Res. (2012) 18:5520–5. doi: 10.1158/1078-0432.CCR-11-2904 PMC3473104 22879387

[61] Johnson LA Clasper S Holt AP Lalor PF Baban D Jackson DG. An inflammation-induced mechanism for leukocyte transmigration across lymphatic vessel endothelium. J Exp Med. (2006) 203:2763–77. doi: 10.1084/jem.20051759 PMC2118156 17116732

[62] Del Prete A Salvi V Soriani A Laffranchi M Sozio F Bosisio D. Dendritic cell subsets in cancer immunity and tumor antigen sensing. Cell Mol Immunol. (2023) 20:432–47. doi: 10.1038/s41423-023-00990-6 PMC10203372 36949244

[63] Zhang Y Ji S Miao G Du S Wang H Yang X. The current role of dendritic cells in the progression and treatment of colorectal cancer. Cancer Biol Med. (2024) 21(9):20240188. doi: 10.20892/j.issn.2095-3941.2024.0188 PMC11414224 39177125

[64] Ascic E Åkerström F Sreekumar Nair M Rosa A Kurochkin I Zimmermannova O. *In vivo* dendritic cell reprogramming for cancer immunotherapy. Science. (2024) 386:eadn9083. doi: 10.1126/science.adn9083 39236156 PMC7616765

[65] Asselin-Labat ML Ruhland MK Ferris ST. Editorial: Antigen presentation in cancer immune responses. Front Immunol. (2025) 16:1558249. doi: 10.3389/fimmu.2025.1558249 39931066 PMC11808280

[66] Gupta YH Khanom A Acton SE. Control of dendritic cell function within the tumour microenvironment. Front Immunol. (2022) 13:733800. doi: 10.3389/fimmu.2022.733800 35355992 PMC8960065

[67] Di Blasio S Van Wigcheren GF Becker A Van Duffelen A Gorris M Verrijp K. The tumour microenvironment shapes dendritic cell plasticity in a human organotypic melanoma culture. Nat Commun. (2020) 11:2749. doi: 10.1038/s41467-020-16583-0 32488012 PMC7265463

[68] Xiao Z Wang R Wang X Yang H Dong J He X. Impaired function of dendritic cells within the tumor microenvironment. Front Immunol. (2023) 14. doi: 10.3389/fimmu.2023.1213629 PMC10333501 37441069

[69] Lim AR Rathmell WK Rathmell JC. The tumor microenvironment as a metabolic barrier to effector T cells and immunotherapy. eLife. (2020) 9:e55185. doi: 10.7554/eLife.55185 32367803 PMC7200151

[70] Scott EN Gocher AM Workman CJ Vignali D. Regulatory T cells: barriers of immune infiltration into the tumor microenvironment. Front Immunol. (2021) 12. doi: 10.3389/fimmu.2021.702726 PMC8222776 34177968

[71] Kim J-H Kim BS Lee S-K. Regulatory T cells in tumor microenvironment and approach for anticancer immunotherapy. Immune Netw. (2020) 20. doi: 10.4110/in.2020.20.e4 PMC7049587 32158592

[72] Arbones ML Ord DC Ley K Ratech H Maynard-Curry C Otten G. Lymphocyte homing and leukocyte rolling and migration are impaired in L-selectin-deficient mice. Immunity. (1994) 1:247–60. doi: 10.1016/1074-7613(94)90076-0 7534203

[73] Sumagin R Sarelius IH. A role for ICAM-1 in maintenance of leukocyte-endothelial cell rolling interactions in inflamed arterioles. Am J Physiol Heart Circ Physiol. (2007) 293:H2786–2798. doi: 10.1152/ajpheart.00720.2007 17704289

[74] Lopez-Garcia L Castro-Manrreza ME. TNF-alpha and IFN-gamma participate in improving the immunoregulatory capacity of mesenchymal stem/stromal cells: importance of cell-cell contact and extracellular vesicles. Int J Mol Sci. (2021) 22. doi: 10.3390/ijms22179531 PMC8431422 34502453

[75] Yang D Liu J Qian H Zhuang Q. Cancer-associated fibroblasts: from basic science to anticancer therapy. Exp Mol Med. (2023) 55:1322–32. doi: 10.1038/s12276-023-01013-0 PMC10394065 37394578

[76] Nabhan M Egan D Kreileder M Zhernovkov V Timosenko E Slidel T. Deciphering the tumour immune microenvironment cell by cell. Immunooncol Technol. (2023) 18:100383. doi: 10.1016/j.iotech.2023.100383 37234284 PMC10206805

[77] Murdamoothoo D Sun Z Yilmaz A Riegel G Abou-Faycal C Deligne C. Tenascin-C immobilizes infiltrating T lymphocytes through CXCL12 promoting breast cancer progression. EMBO Mol Med. (2021) 13:e13270. doi: 10.15252/emmm.202013270 33988305 PMC8185552

[78] Bottcher JP Reis E Sousa C. The role of type 1 conventional dendritic cells in cancer immunity. Trends Cancer. (2018) 4:784–92. doi: 10.1016/j.trecan.2018.09.001 PMC6207145 30352680

[79] Theisen D Murphy K. The role of cDC1s *in vivo*: CD8 T cell priming through cross-presentation. F1000Res. (2017) 6:98. doi: 10.12688/f1000research 28184299 PMC5288679

[80] Lei X De Groot DC Welters MJP De Wit T Schrama E Van Eenennaam H. CD4(+) T cells produce IFN-I to license cDC1s for induction of cytotoxic T-cell activity in human tumors. Cell Mol Immunol. (2024) 21:374–92. doi: 10.1038/s41423-024-01133-1 PMC10978876 38383773

[81] Ferris ST Durai V Wu R Theisen DJ Ward JP Bern MD. cDC1 prime and are licensed by CD4(+) T cells to induce anti-tumour immunity. Nature. (2020) 584:624–9. doi: 10.1038/s41586-020-2611-3 PMC7469755 32788723

[82] Nayak DA Sedlacek AL Cillo AR Watkins SC Binder RJ. CD91 and Its Ligand gp96 Confer Cross-Priming Capabilities to Multiple APCs during Immune Responses to Nascent, Emerging Tumors. Cancer Immunol Res. (2024) 12:1663–76. doi: 10.1158/2326-6066.CIR-24-0326 PMC11614702 39269437

[83] Diamond MS Lin JH Vonderheide RH. Site-dependent immune escape due to impaired dendritic cell cross-priming. Cancer Immunol Res. (2021) 9:877–90. doi: 10.1158/2326-6066.CIR-20-0785 PMC8655819 34145076

[84] Malissen B Gregoire C Malissen M Roncagalli R. Integrative biology of T cell activation. Nat Immunol. (2014) 15:790–7. doi: 10.1038/ni.2959 25137453

[85] Baharun NB Adam A Zailani M Rajpoot NM Xu Q Zin RRM. Automated scoring methods for quantitative interpretation of Tumour infiltrating lymphocytes (TILs) in breast cancer: a systematic review. BMC Cancer. (2024) 24:1202. doi: 10.1186/s12885-024-12962-8 39350098 PMC11440723

[86] Schlam I Loi S Salgado R Swain SM. Tumor-infiltrating lymphocytes in HER2-positive breast cancer: potential impact and challenges. ESMO Open. (2025) 10:104120. doi: 10.1016/j.esmoop.2024.104120 39826475 PMC11786075

[87] Loi S Michiels S Adams S Loibl S Budczies J Denkert C. The journey of tumor-infiltrating lymphocytes as a biomarker in breast cancer: clinical utility in an era of checkpoint inhibition. Ann Oncol. (2021) 32:1236–44. doi: 10.1016/j.annonc.2021.07.007 34311075

[88] Angelico G Broggi G Tinnirello G Puzzo L Vecchio GM Salvatorelli L. Tumor infiltrating lymphocytes (TILS) and PD-L1 expression in breast cancer: A review of current evidence and prognostic implications from pathologist’s perspective. Cancers (Basel). (2023) 15. doi: 10.3390/cancers15184479 PMC10526828 37760449

[89] Xia ZA Lu C Pan C Li J Li J Mao Y. The expression profiles of signature genes from CD103(+)LAG3(+) tumour-infiltrating lymphocyte subsets predict breast cancer survival. BMC Med. (2023) 21:268. doi: 10.1186/s12916-023-02960-1 37488535 PMC10367329

[90] Ciarka A Piatek M Peksa R Kunc M Senkus E. Tumor-infiltrating lymphocytes (TILs) in breast cancer: prognostic and predictive significance across molecular subtypes. Biomedicines. (2024) 12. doi: 10.3390/biomedicines12040763 PMC11048219 38672117

[91] Radvanyi LG Bernatchez C Zhang M Fox PS Miller P Chacon J. Specific lymphocyte subsets predict response to adoptive cell therapy using expanded autologous tumor-infiltrating lymphocytes in metastatic melanoma patients. Clin Cancer Res. (2012) 18:6758–70. doi: 10.1158/1078-0432.CCR-12-1177 PMC3525747 23032743

[92] Forget MA Haymaker C Hess KR Meng YJ Creasy C Karpinets T. Prospective analysis of adoptive TIL therapy in patients with metastatic melanoma: response, impact of anti-CTLA4, and biomarkers to predict clinical outcome. Clin Cancer Res. (2018) 24:4416–28. doi: 10.1158/1078-0432.CCR-17-3649 PMC6139043 29848573

[93] Dafni U Michielin O Lluesma SM Tsourti Z Polydoropoulou V Karlis D. Efficacy of adoptive therapy with tumor-infiltrating lymphocytes and recombinant interleukin-2 in advanced cutaneous melanoma: a systematic review and meta-analysis. Ann Oncol. (2019) 30:1902–13. doi: 10.1093/annonc/mdz398 31566658

[94] Albarran Fernandez V Ballestin Martinez P Stoltenborg Granhoj J Borch TH Donia M Marie Svane I. Biomarkers for response to TIL therapy: a comprehensive review. J Immunother Cancer. (2024) 12. doi: 10.1136/jitc-2023-008640 PMC10941183 38485186

[95] Seitter SJ Sherry RM Yang JC Robbins PF Shindorf ML Copeland AR. Impact of prior treatment on the efficacy of adoptive transfer of tumor-infiltrating lymphocytes in patients with metastatic melanoma. Clin Cancer Res. (2021) 27:5289–98. doi: 10.1158/1078-0432.CCR-21-1171 PMC8857302 34413159

[96] Madsen CO Velasco Santiago M Martinenaite E Holz Borch T Donia M Svane IM. Peripheral immune biomarkers associated with response to adoptive cell therapy with tumor infiltrating lymphocytes. Clin Exp Immunol. (2025). doi: 10.1093/cei/uxaf010 PMC12010344 39965099

[97] Galon J Costes A Sanchez-Cabo F Kirilovsky A Mlecnik B Lagorce-Pages C. Type, density, and location of immune cells within human colorectal tumors predict clinical outcome. Science. (2006) 313:1960–4. doi: 10.1126/science.1129139 17008531

[98] Tumeh PC Harview CL Yearley JH Shintaku IP Taylor EJ Robert L. PD-1 blockade induces responses by inhibiting adaptive immune resistance. Nature. (2014) 515:568–71. doi: 10.1038/nature13954 PMC4246418 25428505

[99] Blessin NC Li W Mandelkow T Jansen HL Yang C Raedler JB. Prognostic role of proliferating CD8(+) cytotoxic Tcells in human cancers. Cell Oncol (Dordr). (2021) 44:793–803. doi: 10.1007/s13402-021-00601-4 33864611 PMC8338812

[100] Elicora A Yaprak Bayrak B Vural C Sezer HF Uzun Erkal S Metin E. Prognostic significance of T lymphocyte subgroups (CD4 and CD8) in lung cancer patients after neoadjuvant chemotherapy. J Cardiothorac Surg. (2024) 19:113. doi: 10.1186/s13019-024-02596-z 38468248 PMC10926577

[101] Geurts VCM Balduzzi S Steenbruggen TG Linn SC Siesling S Badve SS. Tumor-infiltrating lymphocytes in patients with stage I triple-negative breast cancer untreated with chemotherapy. JAMA Oncol. (2024) 10:1077–86. doi: 10.1001/jamaoncol.2024.1917 PMC11211993 38935352

[102] Yan Q Li S He L Chen N. Prognostic implications of tumor-infiltrating lymphocytes in non-small cell lung cancer: a systematic review and meta-analysis. Front Immunol. (2024) 15:1476365. doi: 10.3389/fimmu.2024.1476365 39372398 PMC11449740

[103] Mariathasan S Turley SJ Nickles D Castiglioni A Yuen K Wang Y. TGFbeta attenuates tumour response to PD-L1 blockade by contributing to exclusion of T cells. Nature. (2018) 554:544–8. doi: 10.1038/nature25501 PMC6028240 29443960

[104] Spathas N Goussia AC Koliou GA Gogas H Zagouri F Batistatou A. Association between CD8+ Tumor infiltrating lymphocytes and the clinical outcome of patients with operable breast cancer treated with adjuvant dose-dense chemotherapy-A 10 year follow-up report of a hellenic cooperative oncology group observational study. Cancers (Basel). (2022) 14. doi: 10.3390/cancers14225635 PMC9688913 36428728

[105] Lotfinejad P Asghari Jafarabadi M Abdoli Shadbad M Kazemi T Pashazadeh F Sandoghchian Shotorbani S. Prognostic role and clinical significance of tumor-infiltrating lymphocyte (TIL) and programmed death ligand 1 (PD-L1) expression in triple-negative breast cancer (TNBC): A systematic review and meta-analysis study. Diagnostics (Basel). (2020) 10. doi: 10.3390/diagnostics10090704 PMC7554852 32957579

[106] Huh JW Lee JH Kim HR. Prognostic significance of tumor-infiltrating lymphocytes for patients with colorectal cancer. Arch Surg. (2012) 147:366–72. doi: 10.1001/archsurg.2012.35 22508783

[107] Denkert C Von Minckwitz G Darb-Esfahani S Lederer B Heppner BI Weber KE. Tumour-infiltrating lymphocytes and prognosis in different subtypes of breast cancer: a pooled analysis of 3771 patients treated with neoadjuvant therapy. Lancet Oncol. (2018) 19:40–50. doi: 10.1016/S1470-2045(17)30904-X 29233559

[108] Fridman WH Pages F Sautes-Fridman C Galon J. The immune contexture in human tumours: impact on clinical outcome. Nat Rev Cancer. (2012) 12:298–306. doi: 10.1038/nrc3245 22419253

[109] Savas P Salgado R Denkert C Sotiriou C Darcy PK Smyth MJ. Clinical relevance of host immunity in breast cancer: from TILs to the clinic. Nat Rev Clin Oncol. (2016) 13:228–41. doi: 10.1038/nrclinonc.2015.215 26667975

[110] Wherry EJ Kurachi M. Molecular and cellular insights into T cell exhaustion. Nat Rev Immunol. (2015) 15:486–99. doi: 10.1038/nri3862 PMC4889009 26205583

[111] Hodi FS O’day SJ Mcdermott DF Weber RW Sosman JA Haanen JB. Improved survival with ipilimumab in patients with metastatic melanoma. N Engl J Med. (2010) 363:711–23. doi: 10.1056/NEJMoa1003466 PMC3549297 20525992

[112] Robert C Schachter J Long GV Arance A Grob JJ Mortier L. Pembrolizumab versus ipilimumab in advanced melanoma. N Engl J Med. (2015) 372:2521–32. doi: 10.1056/NEJMoa1503093 25891173

[113] Li D Shao F Yu Q Wu R Tuo Z Wang J. The complex interplay of tumor-infiltrating cells in driving therapeutic resistance pathways. Cell Commun Signal. (2024) 22:405. doi: 10.1186/s12964-024-01776-7 39160622 PMC11331645

[114] Principe N Phung AL Stevens KLP Elaskalani O Wylie B Tilsed CM. Anti-metabolite chemotherapy increases LAG-3 expressing tumor-infiltrating lymphocytes which can be targeted by combination immune checkpoint blockade. J Immunother Cancer. (2024) 12. doi: 10.1136/jitc-2023-008568 PMC11440230 39343508

[115] Betof Warner A Hamid O Komanduri K Amaria R Butler MO Haanen J. Expert consensus guidelines on management and best practices for tumor-infiltrating lymphocyte cell therapy. J Immunother Cancer. (2024) 12. doi: 10.1136/jitc-2023-008735 PMC11005706 38423748

[116] Herbst RS Soria JC Kowanetz M Fine GD Hamid O Gordon MS. Predictive correlates of response to the anti-PD-L1 antibody MPDL3280A in cancer patients. Nature. (2014) 515:563–7. doi: 10.1038/nature14011 PMC4836193 25428504

[117] Yu P Fu YX. Tumor-infiltrating T lymphocytes: friends or foes? Lab Invest. (2006) 86:231–45. doi: 10.1038/labinvest.3700389 16446705

[118] Dakal TC George N Xu C Suravajhala P Kumar A. Predictive and prognostic relevance of tumor-infiltrating immune cells: tailoring personalized treatments against different cancer types. Cancers (Basel). (2024) 16. doi: 10.3390/cancers16091626 PMC11082991 38730579

[119] Chraa D Naim A Olive D Badou A. T lymphocyte subsets in cancer immunity: Friends or foes. J Leukoc Biol. (2019) 105:243–55. doi: 10.1002/JLB.MR0318-097R 30387907

[120] Wu B Zhang B Li B Wu H Jiang M. Cold and hot tumors: from molecular mechanisms to targeted therapy. Signal Transduct Target Ther. (2024) 9:274. doi: 10.1038/s41392-024-01979-x 39420203 PMC11491057

[121] Granhøj JS Witness Præst Jensen A Presti M Met Ö Svane IM Donia M. Tumor-infiltrating lymphocytes for adoptive cell therapy: recent advances, challenges, and future directions. Expert Opin Biol Ther. (2022) 22(5):627–641. doi: 10.1080/14712598.2022.2064711 35414331

[122] Jiang P Gu S Pan D Fu J Sahu A Hu X. Signatures of T cell dysfunction and exclusion predict cancer immunotherapy response. Nat Med. (2018) 24:1550–8. doi: 10.1038/s41591-018-0136-1 PMC6487502 30127393

[123] Ye J Livergood RS Peng G. The role and regulation of human Th17 cells in tumor immunity. Am J Pathol. (2013) 182:10–20. doi: 10.1016/j.ajpath.2012.08.041 23159950 PMC3532708

[124] Zhang Y Zhu K Wang X Zhao Y Shi J Liu Z. Roles of IL-4, IL-13, and their receptors in lung cancer. J Interferon Cytokine Res. (2024) 44:399–407. doi: 10.1089/jir.2024.0008 38516928

[125] Li C Jiang P Wei S Xu X Wang J. Regulatory T cells in tumor microenvironment: new mechanisms, potential therapeutic strategies and future prospects. Mol Cancer. (2020) 19:116. doi: 10.1186/s12943-020-01234-1 32680511 PMC7367382

[126] Zeng G Jin L Ying Q Chen H Thembinkosi MC Yang C. Regulatory T cells in cancer immunotherapy: basic research outcomes and clinical directions. Cancer Manag Res. (2020) 12:10411–21. doi: 10.2147/CMAR.S265828 PMC7586057 33116895

[127] Dabrowska A Grubba M Balihodzic A Szot O Sobocki BK Perdyan A. The role of regulatory T cells in cancer treatment resistance. Int J Mol Sci. (2023) 24. doi: 10.3390/ijms241814114 PMC10531820 37762416

[128] Levental KR Yu H Kass L Lakins JN Egeblad M Erler JT. Matrix crosslinking forces tumor progression by enhancing integrin signaling. Cell. (2009) 139:891–906. doi: 10.1016/j.cell.2009.10.027 19931152 PMC2788004

[129] Kohli K Pillarisetty VG Kim TS. Key chemokines direct migration of immune cells in solid tumors. Cancer Gene Ther. (2022) 29:10–21. doi: 10.1038/s41417-021-00303-x 33603130 PMC8761573

[130] Gajewski TF Woo SR Zha Y Spaapen R Zheng Y Corrales L. Cancer immunotherapy strategies based on overcoming barriers within the tumor microenvironment. Curr Opin Immunol. (2013) 25:268–76. doi: 10.1016/j.coi.2013.02.009 23579075

[131] Rizvi NA Hellmann MD Snyder A Kvistborg P Makarov V Havel JJ. Cancer immunology. Mutational landscape determines sensitivity to PD-1 blockade in non-small cell lung cancer. Science. (2015) 348:124–8. doi: 10.1126/science.aaa1348 PMC4993154 25765070

[132] Paulson KG Voillet V Mcafee MS Hunter DS Wagener FD Perdicchio M. Acquired cancer resistance to combination immunotherapy from transcriptional loss of class I HLA. Nat Commun. (2018) 9:3868. doi: 10.1038/s41467-018-06300-3 30250229 PMC6155241

[133] Tie Y Tang F Wei YQ Wei XW. Immunosuppressive cells in cancer: mechanisms and potential therapeutic targets. J Hematol Oncol. (2022) 15:61. doi: 10.1186/s13045-022-01282-8 35585567 PMC9118588

[134] Zhang W Wang M Ji C Liu X Gu B Dong T. Macrophage polarization in the tumor microenvironment: Emerging roles and therapeutic potentials. BioMed Pharmacother. (2024) 177:116930. doi: 10.1016/j.biopha.2024.116930 38878638

[135] Wang S Wang J Chen Z Luo J Guo W Sun L. Targeting M2-like tumor-associated macrophages is a potential therapeutic approach to overcome antitumor drug resistance. NPJ Precis Oncol. (2024) 8:31. doi: 10.1038/s41698-024-00522-z 38341519 PMC10858952

[136] Huang R Kang T Chen S. The role of tumor-associated macrophages in tumor immune evasion. J Cancer Res Clin Oncol. (2024) 150:238. doi: 10.1007/s00432-024-05777-4 38713256 PMC11076352

[137] Huang J Zhao Y Zhao K Yin K Wang S. Function of reactive oxygen species in myeloid-derived suppressor cells. Front Immunol. (2023) 14:1226443. doi: 10.3389/fimmu.2023.1226443 37646034 PMC10461062

[138] Mirjacic Martinovic K Vuletic A Malisic E Srdic-Rajic T Tisma Miletic N Babovic N. Increased circulating TGF-beta1 is associated with impairment in NK cell effector functions in metastatic melanoma patients. Growth Factors. (2022) 40:231–9. doi: 10.1177/17534259231172079 36129407

[139] De Sanctis F Adamo A Cane S Ugel S. Targeting tumour-reprogrammed myeloid cells: the new battleground in cancer immunotherapy. Semin Immunopathol. (2023) 45:163–86. doi: 10.1007/s00281-022-00965-1 PMC9513014 36161514

[140] Tobin RP Jordan KR Kapoor P Spongberg E Davis D Vorwald VM. IL-6 and IL-8 are linked with myeloid-derived suppressor cell accumulation and correlate with poor clinical outcomes in melanoma patients. Front Oncol. (2019) 9:1223. doi: 10.3389/fonc.2019.01223 31781510 PMC6857649

[141] Facciabene A Peng X Hagemann IS Balint K Barchetti A Wang LP. Tumour hypoxia promotes tolerance and angiogenesis via CCL28 and T(reg) cells. Nature. (2011) 475:226–30. doi: 10.1038/nature10169 21753853

[142] Salminen A Kaarniranta K Kauppinen A. Insulin/IGF-1 signaling promotes immunosuppression via the STAT3 pathway: impact on the aging process and age-related diseases. Inflammation Res. (2021) 70:1043–61. doi: 10.1007/s00011-021-01498-3 PMC8572812 34476533

[143] Zhang Y Gao C Cao F Wu Y Chen S Han X. Pan-cancer analysis of IGF-1 and IGF-1R as potential prognostic biomarkers and immunotherapy targets. Front Oncol. (2021) 11:755341. doi: 10.3389/fonc.2021.755341 34804946 PMC8602838

[144] Mollavelioglu B Cetin Aktas E Cabioglu N Abbasov A Onder S Emiroglu S. High co-expression of immune checkpoint receptors PD-1, CTLA-4, LAG-3, TIM-3, and TIGIT on tumor-infiltrating lymphocytes in early-stage breast cancer. World J Surg Oncol. (2022) 20:349. doi: 10.1186/s12957-022-02810-z 36271406 PMC9587596

[145] Emami Nejad A Najafgholian S Rostami A Sistani A Shojaeifar S Esparvarinha M. The role of hypoxia in the tumor microenvironment and development of cancer stem cell: a novel approach to developing treatment. Cancer Cell Int. (2021) 21:62. doi: 10.1186/s12935-020-01719-5 33472628 PMC7816485

[146] Zhang S Zhang X Yang H Liang T Bai X. Hurdle or thruster: Glucose metabolism of T cells in anti-tumour immunity. Biochim Biophys Acta Rev Cancer. (2024) 1879:189022. doi: 10.1016/j.bbcan.2023.189022 37993001

[147] Rodriguez PC Ochoa AC. Arginine regulation by myeloid derived suppressor cells and tolerance in cancer: mechanisms and therapeutic perspectives. Immunol Rev. (2008) 222:180–91. doi: 10.1111/j.1600-065X.2008.00608.x PMC3546504 18364002

[148] Wang JX Choi SYC Niu X Kang N Xue H Killam J. Lactic acid and an acidic tumor microenvironment suppress anticancer immunity. Int J Mol Sci. (2020) 21. doi: 10.3390/ijms21218363 PMC7664620 33171818

[149] Shah R Ibis B Kashyap M Boussiotis VA. The role of ROS in tumor infiltrating immune cells and cancer immunotherapy. Metabolism. (2024) 151:155747. doi: 10.1016/j.metabol.2023.155747 38042522 PMC10872310

[150] Guo J Wang C Luo N Wu Y Huang W Zhu J. IL-2-free tumor-infiltrating lymphocyte therapy with PD-1 blockade demonstrates potent efficacy in advanced gynecologic cancer. BMC Med. (2024) 22:207. doi: 10.1186/s12916-024-03420-0 38769543 PMC11106999

[151] Schlabach MR Lin S Collester ZR Wrocklage C Shenker S Calnan C. Rational design of a SOCS1-edited tumor-infiltrating lymphocyte therapy using CRISPR/Cas9 screens. J Clin Invest. (2023) 133. doi: 10.1101/2023.09.05.555798 PMC10721144 38099496

[152] Liu Y Sun J Sheng Y Wang J Jin J Li F. Abstract 4062: The discovery and development of a CRISPR/Cas9-engineered tumor-infiltrating lymphocytes product (GT316) as a next-generation TIL therapy. Cancer Res. (2023) 83:4062–2. doi: 10.1158/1538-7445.AM2023-4062

[153] Zhou S Zhu M Meng F Shao J Xu Q Wei J. Evaluation of PD-1 blockade using a multicellular tumor spheroid model. Am J Transl Res. (2019) 11:7471–8. PMC6943460 31934294

[154] Liu Z Li Z. Molecular imaging in tracking tumor-specific cytotoxic T lymphocytes (CTLs). Theranostics. (2014) 4:990–1001. doi: 10.7150/thno.9268 25157278 PMC4142291

[155] Moore N Doty D Zielstorff M Kariv I Moy LY Gimbel A. A multiplexed microfluidic system for evaluation of dynamics of immune-tumor interactions. Lab Chip. (2018) 18:1844–58. doi: 10.1039/C8LC00256H 29796561

[156] Datta P Dey M Ataie Z Unutmaz D Ozbolat IT. 3D bioprinting for reconstituting the cancer microenvironment. NPJ Precis Oncol. (2020) 4:18. doi: 10.1038/s41698-020-0121-2 32793806 PMC7385083

[157] Flores-Torres S Dimitriou NM Pardo LA Kort-Mascort J Pal S Peza-Chavez O. Bioprinted multicomponent hydrogel co-culture tumor-immune model for assessing and simulating tumor-infiltrated lymphocyte migration and functional activation. ACS Appl Mater Interfaces. (2023) 15:33250–62. doi: 10.1021/acsami.3c02995 37404007

[158] Kingsley DM Roberge CL Rudkouskaya A Faulkner DE Barroso M Intes X. Laser-based 3D bioprinting for spatial and size control of tumor spheroids and embryoid bodies. Acta Biomater. (2019) 95:357–70. doi: 10.1016/j.actbio.2019.02.014 PMC7171976 30776506

[159] Clarke SR Barnden M Kurts C Carbone FR Miller JF Heath WR. Characterization of the ovalbumin-specific TCR transgenic line OT-I: MHC elements for positive and negative selection. Immunol Cell Biol. (2000) 78:110–7. doi: 10.1046/j.1440-1711.2000.00889.x 10762410

[160] Wong K Wrocklage C Lin S Mercier IL Bullock C Cadzow L. 204 KSQ-004: Unbiased pair-wise discovery of SOCS1 and Regnase-1 as the top CRISPR/Cas9 dual-edit combination enhancing *in vivo* TIL potency against solid tumors. J ImmunoTherapy Cancer. (2021) 9:A215–5. doi: 10.1136/jitc-2021-SITC2021.204

[161] Shields NJ Peyroux EM Ferguson AL Steain M Neumann S Young SL. Late-stage MC38 tumours recapitulate features of human colorectal cancer - implications for appropriate timepoint selection in preclinical studies. Front Immunol. (2023) 14:1152035. doi: 10.3389/fimmu.2023.1152035 37153625 PMC10160415

[162] Park I Hong S Seok J Lucia SE Song E Kim M. Longitudinal intravital imaging of tumor-infiltrating lymphocyte motility in breast cancer models. J Breast Cancer. (2021) 24:463–73. doi: 10.4048/jbc.2021.24.e40 PMC8561133 34652077

[163] Horowitz NB Mohammad I Moreno-Nieves UY Koliesnik I Tran Q Sunwoo JB. Humanized mouse models for the advancement of innate lymphoid cell-based cancer immunotherapies. Front Immunol. (2021) 12:648580. doi: 10.3389/fimmu.2021.648580 33968039 PMC8100438

[164] Orecchioni S Falvo P Talarico G Mitola G Bravetti G Mancuso P. Vinorelbine and intermittent cyclophosphamide sensitize an aggressive myc-driven B-cell lymphoma to anti-PD-1 by an immunological memory effective against tumor re-challenge. J Clin Med. (2023) 12. doi: 10.3390/jcm12072535 PMC10095342 37048617

[165] Sun Q Hong Z Zhang C Wang L Han Z Ma D. Immune checkpoint therapy for solid tumours: clinical dilemmas and future trends. Signal Transduct Target Ther. (2023) 8:320. doi: 10.1038/s41392-023-01522-4 37635168 PMC10460796

[166] Palmer DC Webber BR Patel Y Johnson MJ Kariya CM Lahr WS. Internal checkpoint regulates T cell neoantigen reactivity and susceptibility to PD1 blockade. Med. (2022) 3:682–704.e688. doi: 10.1016/j.medj.2022.07.008 36007524 PMC9847506

[167] Chacon JA Wu RC Sukhumalchandra P Molldrem JJ Sarnaik A Pilon-Thomas S. Co-stimulation through 4-1BB/CD137 improves the expansion and function of CD8(+) melanoma tumor-infiltrating lymphocytes for adoptive T-cell therapy. PLoS One. (2013) 8:e60031. doi: 10.1371/journal.pone.0060031 23560068 PMC3613355

[168] Varayathu H Sarathy V Thomas BE Mufti SS Naik R. Combination strategies to augment immune check point inhibitors efficacy - implications for translational research. Front Oncol. (2021) 11:559161. doi: 10.3389/fonc.2021.559161 34123767 PMC8193928

[169] Chelvanambi M Fecek RJ Taylor JL Storkus WJ. STING agonist-based treatment promotes vascular normalization and tertiary lymphoid structure formation in the therapeutic melanoma microenvironment. J Immunother Cancer. (2021) 9. doi: 10.1136/jitc-2020-001906 PMC7852948 33526609

[170] Mowat C Mosley SR Namdar A Schiller D Baker K. Anti-tumor immunity in mismatch repair-deficient colorectal cancers requires type I IFN-driven CCL5 and CXCL10. J Exp Med. (2021) 218. doi: 10.1084/jem.20210108 PMC8313406 34297038

[171] Tian Y Xie D Yang L. Engineering strategies to enhance oncolytic viruses in cancer immunotherapy. Signal Transduct Target Ther. (2022) 7:117. doi: 10.1038/s41392-022-00951-x 35387984 PMC8987060

[172] Yin ZS Wang Z. Strategies for engineering oncolytic viruses to enhance cancer immunotherapy. Front Pharmacol. (2024) 15:1450203. doi: 10.3389/fphar.2024.1450203 39309012 PMC11413971

[173] Wang X Venet D Lifrange F Larsimont D Rediti M Stenbeck L. Spatial transcriptomics reveals substantial heterogeneity in triple-negative breast cancer with potential clinical implications. Nat Commun. (2024) 15:10232. doi: 10.1038/s41467-024-54145-w 39592577 PMC11599601

[174] Huber F Arnaud M Stevenson BJ Michaux J Benedetti F Thevenet J. A comprehensive proteogenomic pipeline for neoantigen discovery to advance personalized cancer immunotherapy. Nat Biotechnol. (2024). doi: 10.1038/s41587-024-02420-y PMC12339364 39394480

[175] Skirzynska A Xue C Shoichet MS. Engineering biomaterials to model immune-tumor interactions *in vitro* . Advanced Materials. (2024) 36:2310637. doi: 10.1002/adma.202310637 38349174

[176] Feng Y Tang Q Wang B Yang Q Zhang Y Lei L. Targeting the tumor microenvironment with biomaterials for enhanced immunotherapeutic efficacy. J Nanobiotechnology. (2024) 22:737. doi: 10.1186/s12951-024-03005-2 39605063 PMC11603847

[177] Xiao M Tang Q Zeng S Yang Q Yang X Tong X. Emerging biomaterials for tumor immunotherapy. Biomaterials Res. (2023) 27:47. doi: 10.1186/s40824-023-00369-8 PMC10189985 37194085

[178] Inamdar VV Hao S Stephan SB Stephan MT. Biomaterial-based scaffolds for direct in *situ* programming of tumor-infiltrating T lymphocytes. J Controlled Release. (2024) 370:310–7. doi: 10.1016/j.jconrel.2024.04.040 38677524

[179] Wang S Sun J Chen K Ma P Lei Q Xing S. Perspectives of tumor-infiltrating lymphocyte treatment in solid tumors. BMC Med. (2021) 19:140. doi: 10.1186/s12916-021-02006-4 34112147 PMC8194199

[180] Zhao Y Deng J Rao S Guo S Shen J Du F. Tumor infiltrating lymphocyte (TIL) therapy for solid tumor treatment: progressions and challenges. Cancers (Basel). (2022) 14. doi: 10.3390/cancers14174160 PMC9455018 36077696

[181] Wu Y Yi M Niu M Mei Q Wu K. Myeloid-derived suppressor cells: an emerging target for anticancer immunotherapy. Mol Cancer. (2022) 21:184. doi: 10.1186/s12943-022-01657-y 36163047 PMC9513992

[182] Natarajan A Veerapathran A Wells A Onimus K Machin M Wardell S. Abstract 2746: Preclinical activity and manufacturing feasibility of genetically modified PDCD-1 knockout (KO) tumor-infiltrating lymphocyte (TIL) cell therapy. Cancer Res. (2022) 82:2746–6. doi: 10.1158/1538-7445.AM2022-2746

[183] Guo J Huang W Zhao B Yu J Cui J Sun J. A first-in-human study of CRISPR/Cas9-engineered tumor infiltrating lymphocytes (TILs) product GT316 as monotherapy in advanced solid tumors. J Clin Oncol. (2024) 42:2549–9. doi: 10.1200/JCO.2024.42.16_suppl.2549

[184] Lin S Martinez G Forget M-A Adlerz K Ghose M Williams L. Abstract 20: KSQ-001EX: An engineered TIL therapy manufactured from a clinical-scale, feeder-free process for the treatment of solid tumor indications. Cancer Res. (2024) 84:20–0. doi: 10.1158/1538-7445.AM2024-20

